# Dietary cobalt oxide nanoparticles alleviate aging through activation of mitochondrial UPR in *Caenorhabditis elegans*

**DOI:** 10.7150/thno.81817

**Published:** 2023-05-27

**Authors:** Wenshu Cong, Yajie Wang, Chunhui Yuan, Mei Xu, Han Wang, You Hu, Xuyan Dai, Yuhua Weng, Peter Timashev, Xing-Jie Liang, Yuanyu Huang

**Affiliations:** 1 Advanced Research Institute of Multidisciplinary Science; School of Life Science; School of Medical Technology; Key Laboratory of Molecular Medicine and Biotherapy; Key Laboratory of Medical Molecule Science and Pharmaceutics Engineering; Beijing Institute of Technology, Beijing 100081, P. R. China.; 2 Jiangsu Key Laboratory of Brain Disease and Bioinformation, Research Center for Biochemistry and Molecular Biology, Xuzhou Medical University, Xuzhou 221004, P. R. China.; 3 Key Laboratory of Combinatorial Biosynthesis and Drug Discovery, Ministry of Education, School of Pharmaceutical Sciences, Wuhan University, Wuhan 430071, P. R. China.; 4 Hunan Agricultural University, Changsha 410128, P. R. China.; 5 Laboratory of Clinical Smart Nanotechnologies, Institute for Regenerative Medicine, Sechenov University, 119991 Moscow, Russia.; 6 CAS Key Laboratory for Biomedical Effects of Nanomaterials and Nanosafety, CAS Center for Excellence in Nanoscience, National Center for Nanoscience and Technology of China, Beijing 100190, P. R. China.

**Keywords:** aging, cobalt oxide nanoparticles, *Caenorhabditis elegans*, mitochondrial, unfolded protein response

## Abstract

Mitochondrial unfolded protein response (UPR^mt^), which is a mitochondrial proteostasis pathway, orchestrates an adaptive reprogramming for metabolism homeostasis and organismal longevity. Similar to other defense systems, compromised UPR^mt^ is a feature of several age-related diseases. Here we report that dimercapto succinic acid (DMSA)-modified cobalt oxide nanoparticles (Co_3_O_4_ NPs), which have received wide-spread attention in biomedical fields, is a promising UPR^mt^ activator and, more importantly, provides a gate for extending healthy lifespan.

**Methods:** UPR^mt^ activation by Co_3_O_4_ NPs was tested in transgenetic *Caenorhabditis elegans* (*C. elegans*) specifically expressing UPR^mt^ reporter Phsp-6::GFP, and the underlying mechanism was further validated by mitochondrial morphology, mtDNA/nDNA, metabolism-related genes' expression, mitonuclear protein imbalance, oyxgen assumption and ATP level in *C. elegans*. Then therapeutic response aganist senescence was monitored by lifespan analysis, lipofusin contents, MDA contents, Fe accumulation, pharyngeal locomotion performance as well as athletic ability (head thrashes and body bends) at different developmental stages of *C. elegans*. RNAi towards *ubl-5* or *atfs-1 in* UPR^mt^ pathway was applied to clarify the role of UPR^mt^ in Co_3_O_4_ NPs -mediated anti-aging effects. Finally, the effect of Co_3_O_4_ NPs on mitochondrial homeostasis and D-galactose-induced cell viability decline in mammalian cells were studied.

**Results:** Co_3_O_4_ NPs was revealed as a bona fide activator of the UPR^mt^ signaling pathway, through fine-tuning mitochondrial dynamics and inducing a stoichiometric imbalance between OXPHOS subunits encoded by mitochondrial DNA (mtDNA) and nuclear DNA (nDNA) at early life stage of* C. elegans.* Phenotypically, Co_3_O_4_ NPs treatment protect *C. elegans* from external stresses. More importantly, dietary low level of Co_3_O_4_ NPs effectively extend lifespan and alleviate aging-related physiological and functional decline of worms, demonstrating its potential roles in delaying aging. While the protective effect exerted by Co_3_O_4_ NPs was compromised in line with *atfs-1* or *ubl-5* RNAi treatment. Further studies verified the conservation of Co_3_O_4_ NPs in activating UPR^mt^ and exerting protective effects in mammalian cells.

**Conclusions:** The results reveal beneficial effects of Co_3_O_4_ NPs on mitochondrial metabolic control, thus presenting their potential efficacy in anti-aging care.

## Introduction

Aging is a main risk factor for diabetes, cancers, cardiovascular diseases and neurodegenerative diseases 1-3. Currently, the global population of aging individuals is growing intensely, which leads to a worldwide increase in longevity expectancy 4, 5. One of the main mechanisms underlying compromised physiological function in aging and age-related diseases is cellular stress and damage 1. Several important protective machineries (i.e., repair and refold machineries and degradation apparatus) have evolved to maintain cellular homeostasis and cope with diverse stresses 6. When these defense machineries are compromised, as observed in aging and age-related diseases, cell function is misregulated and cell death is accelerated 7-10.

Mitochondrial unfolded protein response (UPR^mt^) is a master mitochondrial proteostasis pathway orchestrating an adaptive reprogramming of metabolism for the maintenance of cellular homeostasis and cytoprotection 11-16. Disruption of UPR^mt^ is associated with protein aggregation and is a feature of several age-related diseases 17, 18. Previous reports have proved it is a conserved longevity mechanism in various animal models and a promising therapeutic target 11-16. Disturbed balance between OXPHOS subunits encoded by mtDNA and nDNA, a state we termed mitonuclear protein imbalance, is considered as the key route to activate UPR^mt^ 15. To this end, many genetic approaches, such as constructing loss- or gain-of-function mutations of mitochondrial components, have been adopted to induce mitonuclear protein imbalance and activate UPR^mt^ for alleviating senescence 13, 15. However, most of these methods show unsatisfactory efficacy with limitations including long-term interference in genes, off-target cleavage, and complex manipulation.

As an essential trace element that generally found in Vitamin B12, cobalt, is critical for the physiological functions and cellular metabolism for animals and human beings 19, 20. Severe dietary deficiencies of cobalt elements are linked to adverse health effects 21, 22. Despite the protective effects, traditional cobalt and cobalt compounds have a narrow window between beneficial and toxic effects 23. Recently, because of multiple advantages such as ease in fabrication, unique bioactivities and low toxicity with acceptable bioavailability, the potential of cobalt based nanoparticles as therapeutic and diagnostic agents has been increasingly investigated 24-26. For instance, Co_3_O_4_ NPs were proved with multiple enzyme-like activities in biocatalysis and bioassays 27-29. Appropriate supplementation of Co^III^ (salen) (acac) was revealed to up-regulate neuroprotective proteins and protect cells from oxidative stresses 22. Another study used Co atoms on N-doped carbon as antioxidants to reduce pro-inflammatory cytokine levels and alleviate sepsis 30. Despite significant progress having been made, the potential effects of Co based nanomaterials on mitochondrial metabolism and the subsequent animal aging process have seldom been explored.

**Scheme 1:**
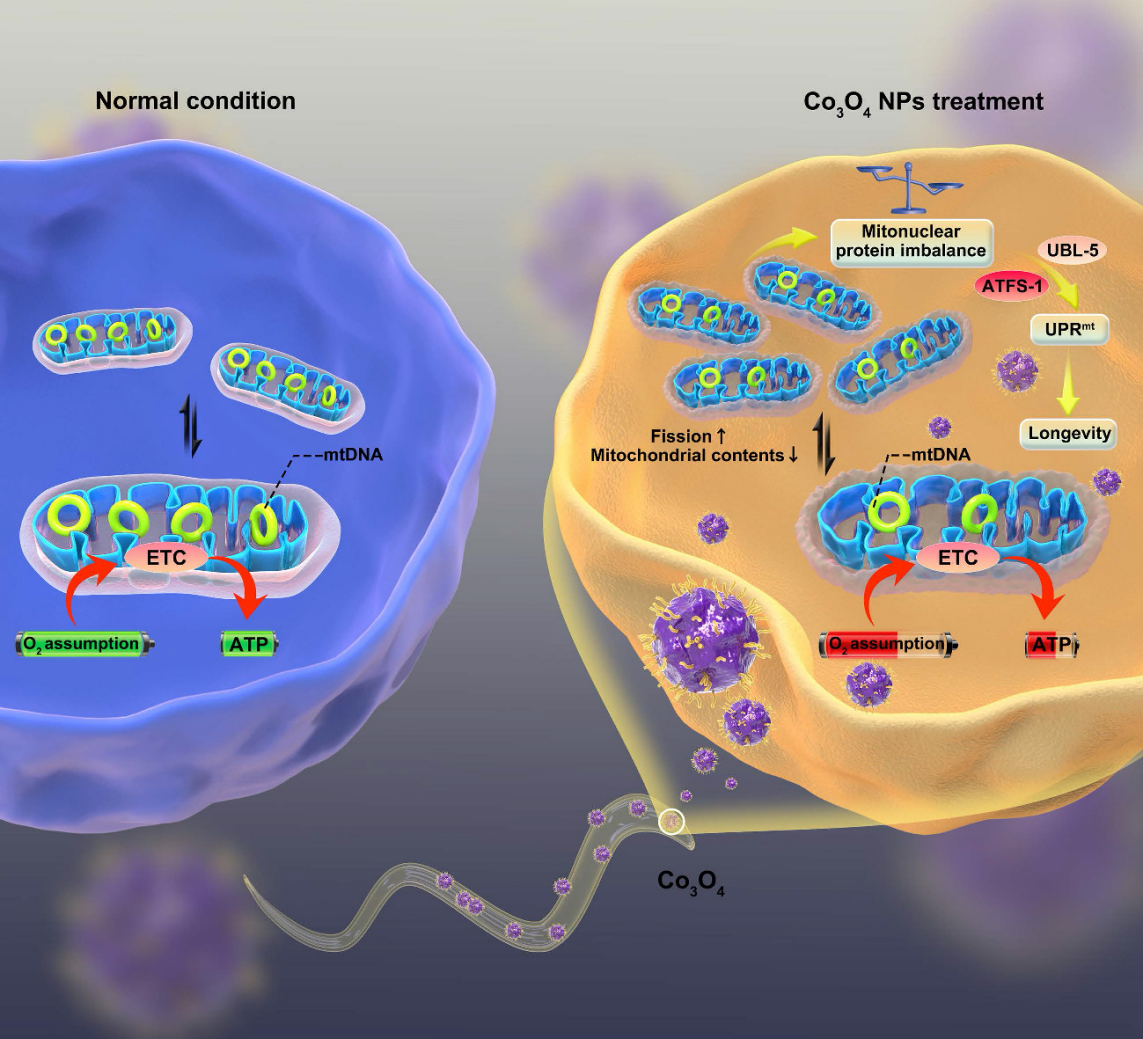
The proposed mechanism by which Co_3_O_4_ NPs shows anti-aging effect on *C. elegans*.

Here, we report that dimercaptosuccinic acid (DMSA) modified cobalt oxide nanoparticles (Co_3_O_4_ NPs) as a mitochondrial UPR^mt^ activator that alleviates the progression of aging (**Scheme 1**). Because of numerous advantages such as short lifespan, similar metabolism routes as in human, conserved genetic information and signaling pathways 31-33, *C. elegans* were adopted as the model animals to conduct *in vivo* study. We firstly confirmed a dosage window for the application of Co_3_O_4_ NPs during the therapy. Then we found that Co_3_O_4_ NPs treatment induce mitonuclear imbalance at the early life stage of worms by influencing fission/fusion and biogensis/degradation balance, followed by UPR^mt^ activation. More importantly, we demonstrated that Co_3_O_4_ NPs treatments extend healthy lifespan in *C. elegans* by stimulating UPR^mt^. Collectively, our results reveal beneficial effects of Co_3_O_4_ NPs on mitochondrial metabolic control, thus presenting their potential efficacy in anti-aging care.

## Results and Discussion

### Biocompatibility and Anti-stress Performance Assessment of Co_3_O_4_ NPs

**Figure 1:**
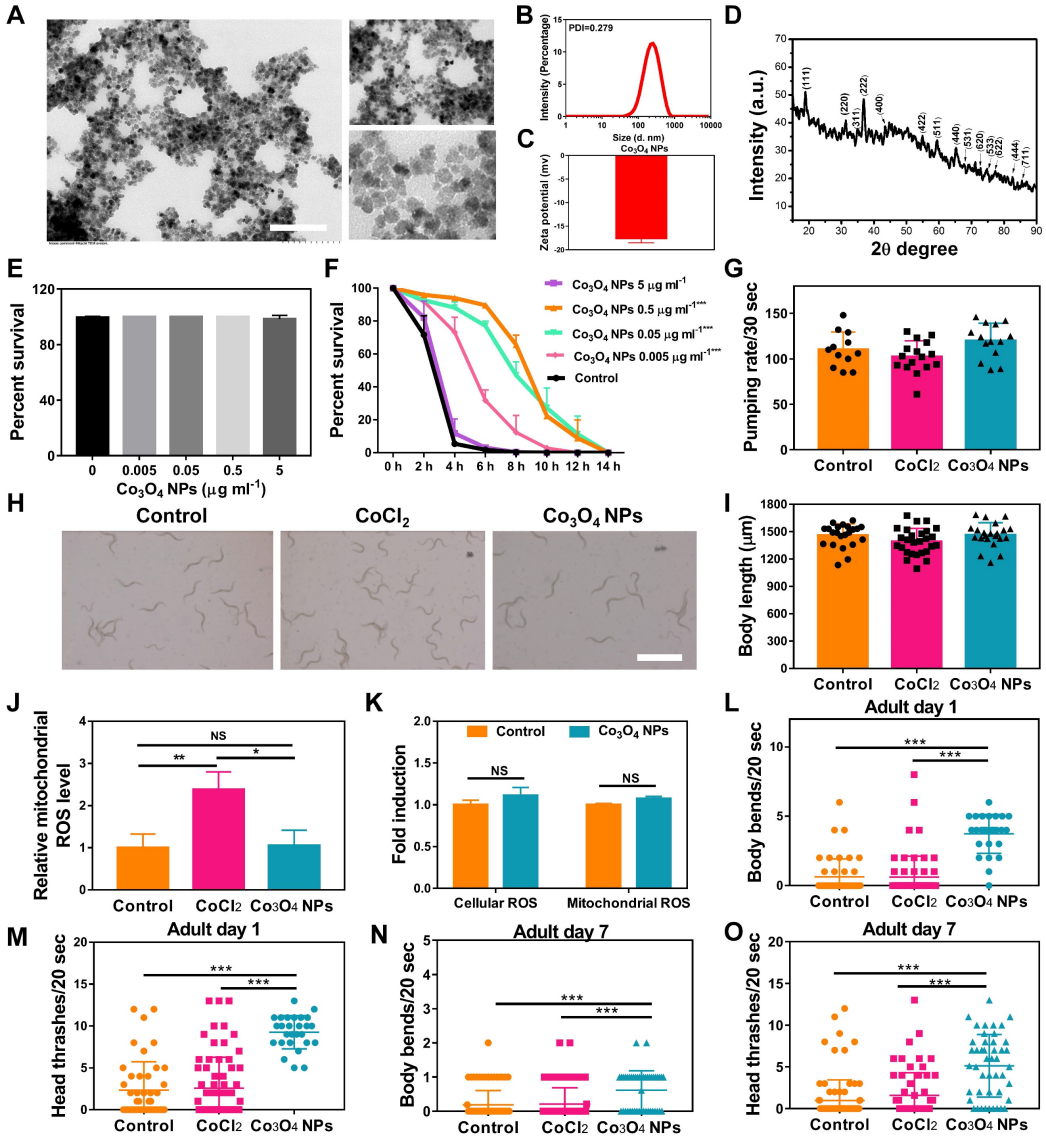
** Characterization of physicochemical properties and biocompatibility evaluation of Co_3_O_4_ NPs.** (A) Representative TEM image of Co_3_O_4_ NPs. Scale bars = 2 μm. DLS histograms (B), zeta potential (C) and powder X-ray diffraction pattern (D) of Co_3_O_4_ NPs. (E) Lethality of worms treated with gradient doses of Co_3_O_4_ NPs treatments. (F) Lifespan of worms treated with Co_3_O_4_ NPs under juglone-induced oxidative stress. Comparison of Co_3_O_4_ NPs and CoCl_2_ at equal doses (0.05 μg mL^-1^) on pumping rate (G) and body length on adult day 1 (H, I). Scale bars = 5 mm. (J) Quantified mitochondrial ROS in *C. elegans* after Co_3_O_4_ NPs and CoCl_2_ treatments. (K) Comparison of mitochondrial and cellular ROS in Co_3_O_4_ NPs and mock-treated worms on adult day 1. Body bend frequency (L) and head thrash frequency (M) of worms on adulthood day 1 under heat stresses; Body bend frequency (N) and head thrash frequency (O) of worms on adulthood day 7 under heat stresses.

The Co_3_O_4_ NPs modified with DMSA used in the study were synthesized by a coprecipitation method as in previous work 26. We first evaluated the physical properties of Co_3_O_4_ NPs. As shown by transmission electron microscopy (TEM), the Co_3_O_4_ NPs were well dispersed and had a uniform spherical shape (**Figure 1A**). The size distributions and zeta potential of the synthesized Co_3_O_4_ NPs were characterized using dynamic light scattering (DLS), which indicated a uniform size of 200 nm and approximately -17.8 eV (**Figure 1B-C**). Then XRD techniques were employed to analyze the morphology and the phase composition. As shown in **Figure 1D**, all the measured diffraction peaks of the Co_3_O_4_ NPs XRD pattern matched well to the standard pattern of hausmannite Co_3_O_4_ NPs, confirming their highly crystalline nature.

To evaluate the biocompatibility of Co_3_O_4_ NPs, wildtype worms at L1 Larva stage were fed with Co_3_O_4_ NPs for three days followed by measurements. We found that Co_3_O_4_ NPs at tested doses (0.005-5 μg mL^-1^) did not induce any adverse effect on survival rate, body length and egg-laying rate in* C. elegans* under normal situations (**Figure 1E and Figure S1**). Interestingly, after the exposure to juglone as oxidative stress stimuli, worms pretreated with Co_3_O_4_ NPs of different concentration at 0.005, 0.05 and 0.5 μg mL^-1^ had an increased lifespan, when compared with blank group, suggesting the dosage-dependent protective effect of Co_3_O_4_ NPs under stress (**Figure 1F**). Since the protective effects of 0.5 μg mL^-1^ and 0.05 μg mL^-1^ under juglone are similar, Co_3_O_4_ NPs at 0.05 μg mL^-1^ concentrations was adopted for the following studies. We continued to compare the biosafety of Co_3_O_4_ NPs versus CoCl_2_. Under normal conditions, it was revealed that neither Co_3_O_4_ NPs nor its inorganic control CoCl_2_ induced any adverse effect on larva in terms of pharyngeal pumping rate and body length in worms (**Figure 1G-I**). In addition, Co_3_O_4_ NPs exposure at 0.05 μg mL^-1^ did not induce significant level of reactive oxygen species (ROS) in both cellular and mitochondrial level evaluated by 2′,7′-dichlorofluorescin diacetate (DCFDA) and Mitosox dyes, correspondingly (**Figure 1J**-**K and Figure S2**). Whearas CoCl_2_ induced ROS to some contents at mitochondrial_,_ reconfirming the superior biosafety of Co_3_O_4_ NPs compared to its inorganic form (**Figure 1J and Figure S2**). Furthermore, both young and aged worms after treated with Co_3_O_4_ NPs appeared more resistant under high temperature induced stressed conditions, as the frequency of body bends and head thrashes remained higher than that of the untreated control groups (**Figure 1L**-**O**). While CoCl_2_ did not show any protective effects (**Figure 1L**-**O**). In conclusion, above findings suggest low levels of Co_3_O_4_ NPs not only have good biocompatibility, but also demonstrate remarkable protective efficacy to *C. elegans* against external stressors. Furthermore, above results also exclude the possibility of dietary intake restriction due to Co_3_O_4_ NPs supplementation 34.

### Co_3_O_4_ NPs Treatment Activates UPR^mt^ Pathway

**Figure 2:**
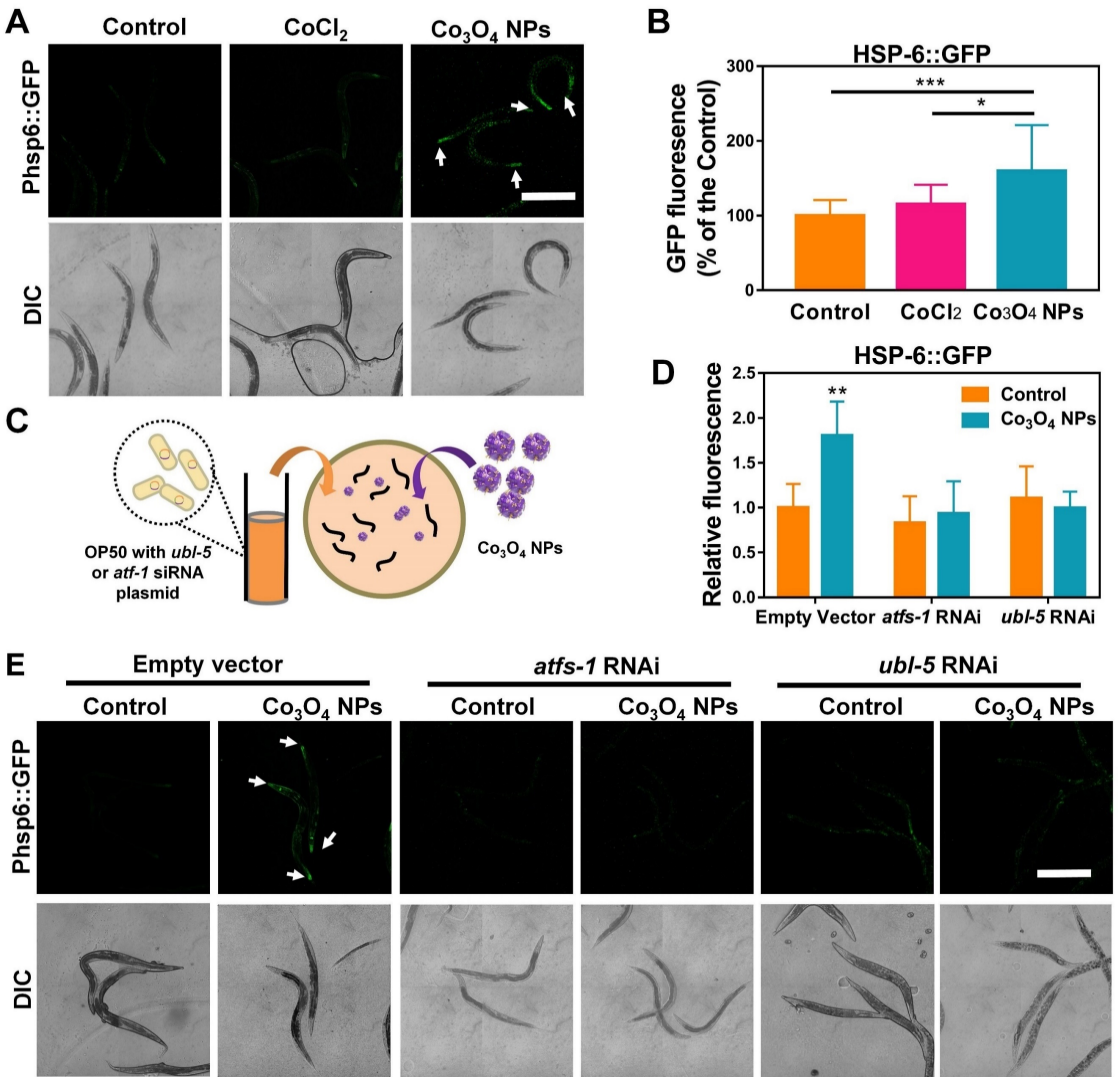
**Effect of Co_3_O_4_ NPs on UPR^mt^ pathway.** Fluorescent images (A) and quantification (B) of UPR^mt^ of worms expressing Phsp-6::GFP treated with Co_3_O_4_ NPs, CoCl_2_ or not. (C) Schematic diagram of the RNAi test procedures. Quantitative results (D) and representative fluorescent pics (E) of *C. elegans* expressing the UPR^mt^ reporter Phsp-6::GFP treated with or without Co_3_O_4_ NPs, in the presence of control dsRNAs or dsRNAs targeting *ubl-5* or *atfs-1*. DIC: differential interference contrast. Worms were synchronized and treated with Co_3_O_4_ NPs from L4 stage until the test days. *P < 0.05, **P < 0.01, ***P < 0.001. Scale bar = 0.5 mm.

The unusual protective effects of Co_3_O_4_ NPs against external stressors inspired us to investigate further. UPR^mt^ is a momentous mitochondrial-to-nuclear stress-signaling pathway that could rewire the metabolic state and improve immune strength 11-16. To figure out the beneficial effect exerted by Co_3_O_4_ NPs, we explored the potential interference of this metabolic sensor by uptake of Co_3_O_4_ NPs. The worms specifically expressing UPR^mt^ reporter Phsp-6::GFP in the mitochondrial were used for *in vivo* monitoring of UPR^mt^ activation after Co_3_O_4_ treatments. Surprisingly, incubation with Co_3_O_4_ NPs robustly upregulated the expression of Phsp-6::GFP by approximately 60%, while CoCl_2_ treatment did not significantly increase GFP signals (**Figure 2A-B**). Similarly, the endogenous transcriptional level of *hsp-6* had been found to be upregulated in the Co_3_O_4_ NPs-treated worms (**Figure S3**). These results confirmed that UPR^mt^ was activated after Co_3_O_4_ NPs treatment. Ubiquitin-Like Protein-5 (UBL-5) and Activating Transcription Factor-1 (ATFS-1) are the two critical UPR^mt^ regulators that activate mitochondrial chaperones and control mitochondrial transcription, respectively 14, 16, 35. To further confirm UPR^mt^ activation, we also fed Co_3_O_4_ NPs to worms with *ubl-5* or *atfs-1* downregulated through *RNAi* (**Figure 2C**). As we expected, the upregulation of Phsp6::GFP activation by Co_3_O_4_ NPs was abolished in the *ubl-5* or *atfs-1* knockdown worms (**Figure 2D-E**), supporting the previous findings that identified Co_3_O_4_ NPs as an activator of the UPR^mt^ pathway.

Additionally, to investigate the role of other pathways involved in stress-resistance paradigm, we also measured the expression of markers of heat shock and ER stress pathways as potential factors for the beneficial effects upon Co_3_O_4_ NPs exposure. Specifically, Co_3_O_4_ NPs did not influence transcriptional expression changes of the transcription factor heat shock factor-1 (HSF-1) which regulates the heat shock response (HSR) or heat shock factor-4 (HSF-4) that regulates endoplasmic reticulum (ER) stress when compared with vehicle control group (**Figure S4**) 36, 37.

### Co_3_O_4_ NPs Treatment Mildly Shift Mitochondrial Homeostasis at Early Life Stages

**Figure 3:**
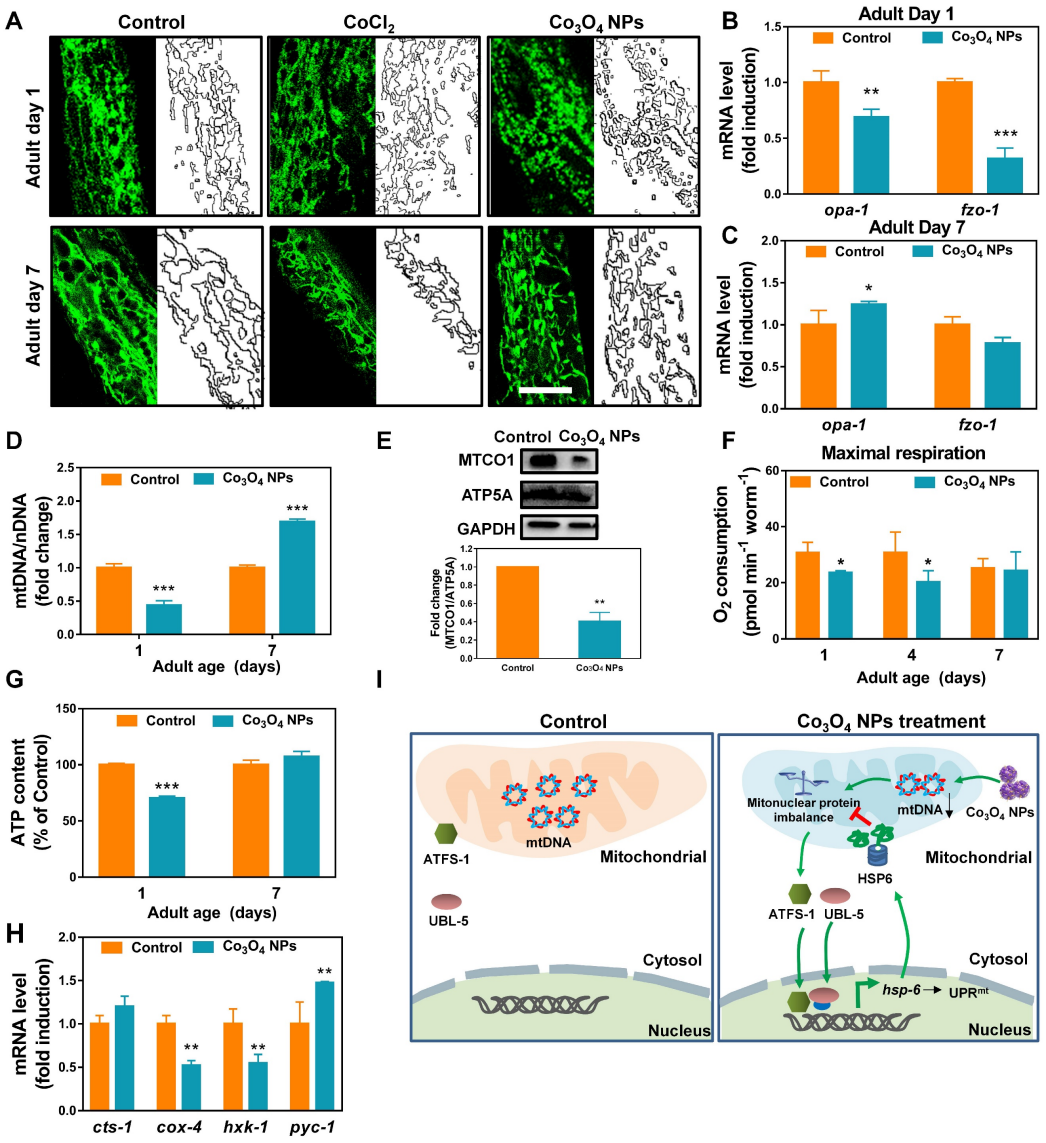
** Effect of Co_3_O_4_ NPs on mitochondrial homeostasis**. (A) Representative GFP images of mitochondrial contents on adult day 1 and 7 of worms expressing mitochondrial-targeted pmyo-3::mito::GFP reporter in body wall muscle. *mRNA* expression of mitochondrial fusion genes *fzo-1* and *opa-1* on adult day 1 (B) and adult day 7 (C). (D) Fold change in mtDNA/nDNA ratio (*nd-1/ act-3*) on days 1 and 7 of adulthood in worms pretreated with Co_3_O_4_ NPs or not. (E) Western blot results and quantitative analysis of OXPHOS subunits encoded by nDNA (ATP5A) and mtDNA (MTCO1) in worms. (F) Comparison of maximum oxygen consumption of N_2_ worms pretreated with Co_3_O_4_ NPs or not. (G) Comparison of ATP levels in N_2_ worms pretreated with Co_3_O_4_ NPs or not. (H) Expression of key metabolic genes* cts-1* (TCA cycle), *hxk-1* (glycolysis), *pyc-1* (gluconeogenesis) and *cox-4* (ETC chain enzyme complex IV) in worms on adult days 1. (I) Scheme summarizing how we hypothesize that Co_3_O_4_ NPs induce activation of UPR^mt^. The bars represent means ± SD. *P < 0.05, **P < 0.01, ***P < 0.001. Scale bar = 10 μm.

Encouraged by the above results, we continued to identify Co_3_O_4_ NPs's mode of action on mitochondrial to explore the mechanism for Co_3_O_4_ NPs induced UPR^mt^ activation. Firstly, transgenic worms expressing mitochondrial-targeted pmyo-3::mito::GFP were used to monitor mitochondrial morphology and contents. It was revealed that the mitochondrial in young worms pretreated with the Co_3_O_4_ NPs appeared to be more fragmented compared to the regular tubular network in the control or CoCl_2_ worms, implying that Co_3_O_4_ NPs promote mitochondrial fission of worms at young stage (**Figure 3A**). While on adult day 7, mitochondrial fragmentation in Co_3_O_4_ NPs treated group disappeared, indicating that nematodes gradually adapted to changes in mitochondrial homeostasis caused by Co_3_O_4_ NPs (**Figure 3A**). Additionally, it was revealed that Co_3_O_4_ NPs treatment downregulated the transcription of the mitochondrial fusion genes *fzo-1* and *opa-1* in young worms (**Figure 3B**). While on adult day 7, *fzo-1* and *opa-1* expression of Co_3_O_4_ NPs treated worms became similar or even higher than untreated controls (**Figure 3C**), which were consistent with the morphological changes of nematodes mitochondrial. Above results indicate that Co_3_O_4_ NPs promote mitochondrial fission at the early life stage, implying the shifted mitochondrial homeostasis caused by Co_3_O_4_ NPs 38.

Disturbed protein homeostasis in mitochondrial is closely related with UPR^mt^ pathway. Typically, mitochondrial proteins are encoded by both nuclear and mitochondrial genomes. Stoichiometric imbalance between the expression of proteins from these two sources, a state we termed mitonuclear protein imbalance, is the key route to activate the mitochondrial unfolded protein response (UPR^mt^) 11, 39-41. To further explore the mechanism by which Co_3_O_4_ NPs treatment activate UPR^mt^, we determined whether Co_3_O_4_ NPs treatment induces mitonuclear protein imbalance. It was found that Co_3_O_4_ NPs treatment reduced the expression of a set of nuclear genes encoding mitochondrial proteins in young adult worms (**Figure S5A**). Moreover, expression of nuclear contents versus mitochondrial contents in terms of mRNA and protein level were measured. According to the results, Co_3_O_4_ NPs treatment decreased the mitochondrial DNA (mtDNA)/nuclear DNA (nDNA) ratios, demonstrating its attenuating effect on mitochondrial biogenesis (**Figure 3D and Figure S5B**). Correspondingly, a stoichiometric mitonuclear protein imbalance verified by the decreased ratio between the mtDNA-encoded MTCO1 and the nDNA-encoded ATP5A as oxidative phosphorylation subunits was found in Co_3_O_4_ NPs treated worms (**Figure 3E**). Together, these data indicated that Co_3_O_4_ NPs treatments decrease mitochondrial abundance and therefore induce mitonuclear imbalance at the early life stage.

Furthermore, we also set out to verify the functional relevance of decreased mitochondrial abundance. It was revealed that the maximum mitochondrial oxygen consumption of nematodes treated with Co_3_O_4_ NPs was slighted down-regulated by 23% and 34% compared to the control groups on adult day 1 and day 4, while appeared unaffected on adult day 7 (**Figure 3F and Figure S6**). And the basal oxygen consumption of young or aged worms appeared not be affected significantly by Co_3_O_4_ NPs (**Figure S7**). In accordance with this, the ATP generation in the Co_3_O_4_ NPs treated nematodes appeared downregulated by about 30% on adult day 1, while became similar to that in the blank control group when worms grew old (**Figure 3G**). Furthermore, the attenuating effect on energy metabolism of Co_3_O_4_ NPs was also confirmed by gene expression analysis of key metabolic enzymes. It was found that Co_3_O_4_ NPs treatment downregulated the gene expression of enzymes controlling key metabolic pathways including the glycolysis genes hexokinase (*hxk-1*) and cytochrome C oxidase IV (*cox-4*). While TCA cycle gene citrate synthase-1 (*cts-1*) remained unchanged and gluconeogenesis gene pyruvate carboxylase (*pyc-1*) were slightly up-regulated, implying that Co_3_O_4_ NPs inhibited carbohydrate catabolism and promoted anabolism in *C. elegans* (**Figure 3H**). Above results demonstrated that Co_3_O_4_ NPs treatment could mildly interefere mitochondrial activities at early life stage, reconfirming the attenuating efficiency of Co_3_O_4_ NPs on mitochondrial abundance.

Collectively, by tracking the changes in mitochondrial morphology, contents and activities of nematodes, we deduced that Co_3_O_4_ NPs activated UPR^mt^ mainly via tuning mitochondrial homeostasis including the fission/fusion and biogensis/degradation balance, followed by inducing mitonuclear imbalance on early life stage (**Figure 3I**). The metabolic homeostasis seems to be restored along with aging, implying the potential adaptive responses. In a different line, mild decrease in mitochondrial contents and acitivies on early life stage exerts beneficial effects on lifespan of worms, flies, and mice 16, 42. Therefore, we speculated that Co_3_O_4_ NPs' regulatory effects on mitochondrial might contribute to its protective effects.

### Co_3_O_4_ NPs Extend *C. elegans* Healthy Lifespan

**Figure 4:**
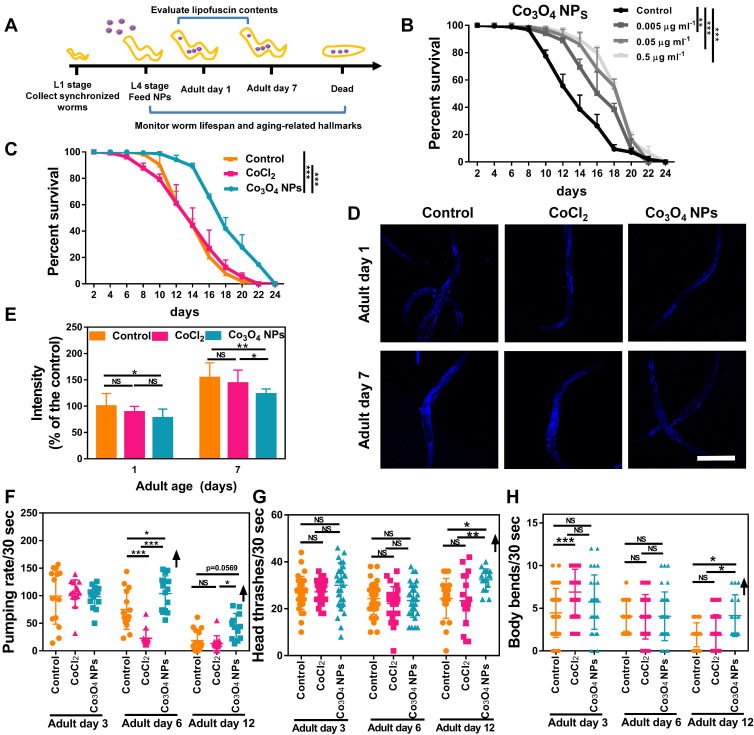
** Co_3_O_4_ NPs extend lifespan and delay age-related behavioral decline in *C. elegans*.** (A) Schematic diagram of the anti-aging experimental procedure. Synchronous *C. elegans* populations were collected and treated with Co_3_O_4_ NPs from the L4 stage until further tests. (B) Lifespan curves of worms in the presence of Co_3_O_4_ NPs at gradient concentrations. (C) Lifespan curves of worms in the presence of Co_3_O_4_ NPs, CoCl_2_ or not. Average lifespan of control group: 15.1 ± 0.7 days; Average lifespan of CoCl_2_ group: 14.8 ± 0.5 days; Average lifespan of Co_3_O_4_ NPs group: 18.6 ± 0.4 days; The representative fluorescence micrographs (D) and average fluorescence intensity (E) of lipofuscin in N_2_ worms on days 1 and 7 of adulthood pretreated with Co_3_O_4_ NPs, CoCl_2_ or not. Pumping rate (F), head thrashes (G) and body bends (H) on days 3, 6 and 12 of adulthood N_2_ worms pretreated with Co_3_O_4_ NPs, CoCl_2_ or not. *P < 0.05, **P < 0.01, ***P < 0.001. Scale bar = 0.5 mm.

Because UPR^mt^ plays a key role in innate immunity and prolongevity machinery in various organisms 11-16, we anticipated an anti-aging role for Co_3_O_4_ NPs in *C. elegans* as well. We first assessed the anti-aging propensity of Co_3_O_4_ NPs by evaluating its efficacy to elongate lifespan of *C. elegans*. Synchronized nematodes were cultured in the medium containing Co_3_O_4_ NPs at different doses of 0.005-0.5 μg mL^-1^ (10-fold increase) until death (**Figure 4A**). As we expected, the worms fed with Co_3_O_4_ NPs showed longer lifespan in a dose-dependent mode, which was approximatedly 1.39 times (with 0.005 μg mL^-1^ Co_3_O_4_ NPs), 1.49 times (with 0.05 μg mL^-1^ Co_3_O_4_ NPs), 1.53 times (with 0.5 μg mL^-1^ Co_3_O_4_ NPs) than that of controls, respectively (**Figure 4B and Figure S8**). It is noteworthy that dietary Co_3_O_4_ NPs with UV-killed OP50 bacteria also prolonged the lifespan of *C. elegans* (**Figure S9A**). Additionally, Co_3_O_4_ NPs at different doses (0.005-0.5 μg mL^-1^) did not affect the growth rate of OP50 bacteria, ruling out bacterial growth rate alteration as the underlying mechanism for Co_3_O_4_ NPs-mediated lifespan extension (**Figure S9B**). To further confirm that the extending effect on lifespan was the direct result of Co_3_O_4_ NPs treatment, we also tested the influence of some other nanoparticles (Fe_2_O_3_ NPs, Mn_3_O_4_ NPs and Prussian NPs) on lifespan. None of these nanomaterials at tested concentrations exerted significant effects on elongating worms' lifespan (**Figure S10**). Moreover, the CoCl_2_ treatment did not extend lifespan of worms, either (**Figure 4C**), which is speculated due to its failure to activate the UPR^mt^ pathway. Above results implied a unique anti-aging feature of Co_3_O_4_ NPs.

We further studied the anti-aging potential of Co_3_O_4_ NPs by monitoring their effects on aging-related biochemical markers and behaviors. Lipofuscin is a non-degradable intralysosomal substance whose accumulation correlates with age and is a useful biomarker for the physiological age of *C. elegan* 43, 44. As shown in **Figure 4D**-**E**, the fluorescence intensity in the nematodes treated with Co_3_O_4_ NPs was less than that of untreated or CoCl_2_ NPs-treated controls on both tested days. Of note, compared with the control group, Co_3_O_4_ NPs-treated aged worms also accumulated lower levels of iron and malondialdehyde (MDA) content, both of which are aging-related markers (**Figure S11**) 45, 46. Furthermore, the pharyngeal locomotion performance was improved on adult days 6, 9 and 12 when the worms were fed with Co_3_O_4_ NPs (**Figure 4F**, **Figure S12 and S13**). We also divided the pharyngeal contractions of worms into three groups: < 6 min^-1^ (not pumping), 6-147 min^-1^ (slow pumping), and >147 min^-1^ (fast pumping), respectively. It was observed that pumping rates decline in the adult nematodes of the control groups along with aging and the behavioral curves of the defined fast pumping worms and low pumping worms intersected on adult day 7. In contrast, the intersection of the curves was delayed until adult day 9 in Co_3_O_4_ NPs group since the fast pumping rate declined more slowly (**Figure S14**). Similar beneficial effects of Co_3_O_4_ NPs on athletic ability indicated by head thrashes and body bends were also observed. As shown in **Figure 4G**-**H**, both head thrashes and body bend frequencies were robustly improved by the Co_3_O_4_ NPs treatment along with aging. Altogether, these data demonstrate the outstanding effects of Co_3_O_4_ NPs on attenuating aging-related behavioral and functional deterioration of *C. elegans*.

### Co_3_O_4_ NPs' Extension of Healthy Lifespan in *C. elegans* is UPR^mt^ Dependent

**Figure 5:**
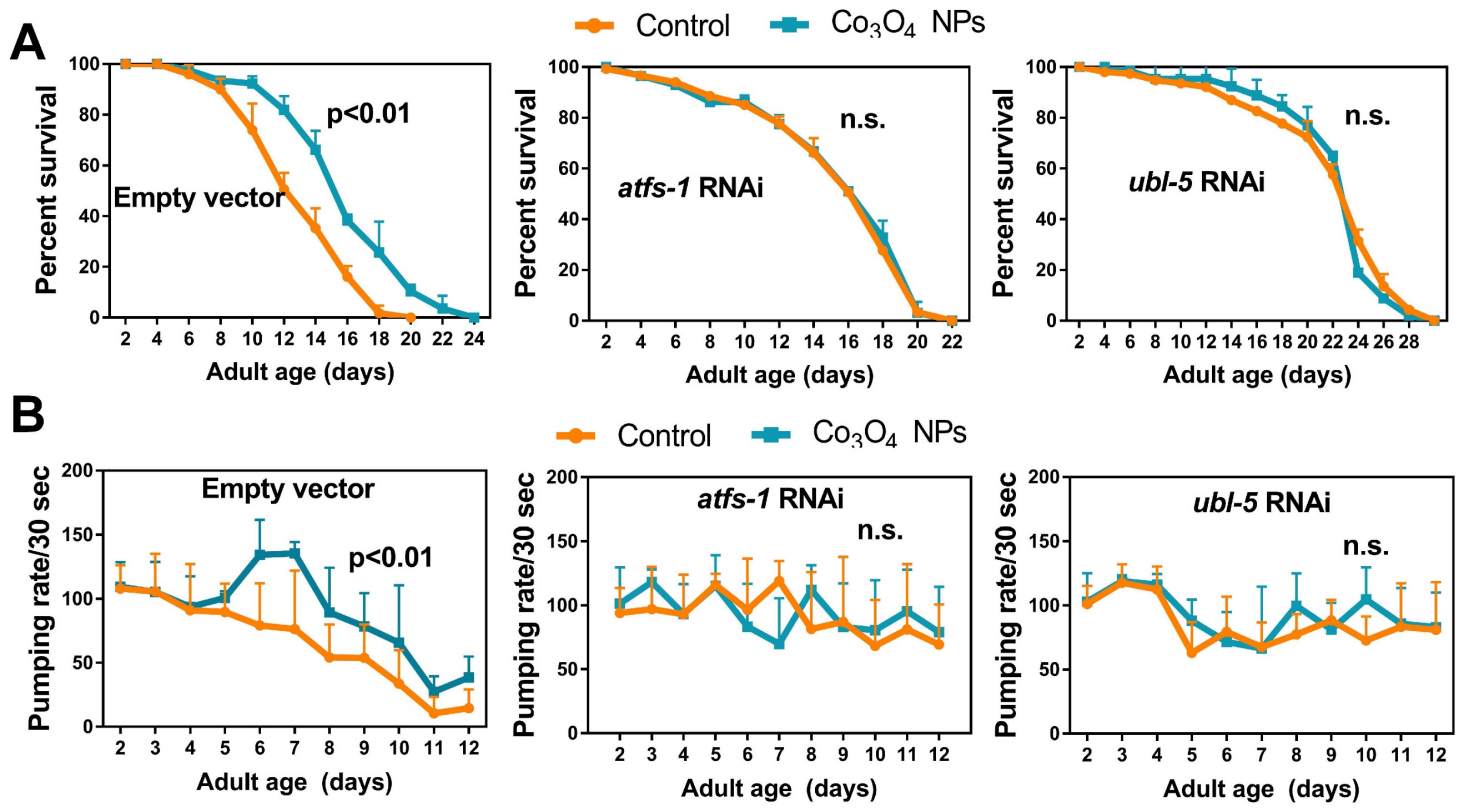
**Co_3_O_4_ NPs confer longevity effects through UPR^mt^.** (A) Lifespan curves of control worms (left), worms treated with* atfs-1* dsRNA (middle) or worms treated with *ubl-5* dsRNAs (right) after Co_3_O_4_ NPs treatments. (B) Age-dependent decline in pharyngeal pumping of control worms (left), worms treated with *atfs-1* dsRNA (middle) or worms treated with *ubl-5* dsRNAs (right) after Co_3_O_4_ NPs treatments.

To further understand the role of UPR^mt^ in Co_3_O_4_ NPs -mediated anti-aging effects, we studied the lifespan of mutants with loss-of-function in *ubl-5*, *atfs-1 or dve-1*, all of which are critical regulators in UPR^mt^ pathway 14, 16, 35. As we expected, attenuating UPR^mt^ by downregulating *ubl-5* or *atfs-1* prevented the lifespan extension induced by Co_3_O_4_ NPs (**Figure 5A**). Supplementation of Co_3_O_4_ NPs does not extend lifespan in the *dve-1 (fx0259)* mutant, either (**Figure S15**). Above observations highlight the importance of UPR^mt^ pathway on the anti-aging efficiency of Co_3_O_4_ NPs. To further confirm that the anti-aging potential was the direct result of UPR^mt^ activation induced by Co_3_O_4_ NPs treatment, we also measured the effect of Co_3_O_4_ NPs on pharyngeal pumping rate in *ubl-5* siRNA, *atfs-1* siRNA or empty vector treated populations. The parameters showed upregulated pharyngeal pumping rate by Co_3_O_4_ NPs treatment was compromised completely or partly in line with *atfs-1* or *ubl-5* RNAi treatment, respectively (**Figure 5B**). Based on above data, we concluded that Co_3_O_4_ NPs promoted longevity and improved aging-related behaviors in *C. elegans* mainly through the activation of UPR^mt^.

### Co_3_O_4_ NPs Treatment Induces UPR^mt^ in Mammalian Cells and Protects Cells from D-galactose-induced Cell Viability Decline

Furthermore, we aimed to investigate Co_3_O_4_ NPs' influence on mitochondrial homeostasis and their protective roles in mammalian cells. Similar with the findings in *C. elegans*, transmission electron microscopy (TEM) analysis of mitochondrial in human embryonic kidney cells (HEK293T) revealed that Co_3_O_4_ NPs treatment promotes cellular mitochondrial fission (**Figure S16A**). Noticeably, mitochondrial fission was also accompanied by compromised mitochondrial biogenesis. Co_3_O_4_ NPs treatment reduced the mtDNA/nDNA ratio (**Figure S16B**), a common marker of mitochondrial abundance in the HEK293T cell line. It was also found that Co_3_O_4_ NPs treatment downregulated the transcriptional expression of a set of mitochondrial proteins in a dose-dependent mode (**Figure S16C**). Consequently, Co_3_O_4_ NPs supplement dose-dependently triggers mitonuclear protein imbalance, reflected by decreased ratio between mtDNA-encoded MTCO1 versus nDNA-encoded SDHA or ATP5A (**Figure S16D-F**). Furthermore, we also demonstrated that the effects of Co_3_O_4_ NPs on UPR^mt^ activation are not unique to *C. elegans*. As shown in **Figure S16D** and **Figure S16G**, Co_3_O_4_ NPs induced the UPR^mt^ protease CLPP at the protein level 47. Consistently, the expression of *hsp60*—the mammalian ortholog of worm *hsp-6*— increased in Co_3_O_4_ NPs treated HEK293T cells (**Figure S16H**). Altogether, these data validate the conservation of the UPR^mt^ signaling pathway induced by Co_3_O_4_ NPs in mammalian cells, as well as the role of mitonuclear protein imbalance therein. Considering the importance of UPR^mt^ in longevity and cellular protection, we next tested the protective efficiency of Co_3_O_4_ NPs in D-galactose-accelerated aging cell models 48. According to CCK8 assay results, the viability of cells exposed to D-galactose was significantly lower than that in blank control group (**Figure S16I**). In contrast, Co_3_O_4_ NPs pretreatment partially reversed D-galactose induced cell viability decline (**Figure S16I**). Altogether, above results indicate the potential of Co_3_O_4_ NPs as UPR^mt^ activator in mammalian systems for anti-aging therapy.

## Conclusions

In summary, our study reveals intrinsic anti-aging effects of Co_3_O_4_ NPs in *C. elegans* models via the activation of the UPR^mt^ signaling pathway. We demonstrate that low levels of Co_3_O_4_ NPs, under the premise of acceptable compatibility in a whole-animal context, could fine-tune mitochondrial dynamics through increasing mitochondrial fission and inducing mitonuclear imbalance during early developmental age for UPR^mt^ activation. We further validate the anti-aging efficacy of Co_3_O_4_ NPs in the *C. elegans* model. Oral administration of Co_3_O_4_ NPs not only extend lifespan but also alleviate aging-related physiological and functional decline. We suggest that activation of UPR^mt^ by Co_3_O_4_ NPs provides a protective mechanism to shield *C. elegans*, otherwise vulnerable in a compromised environment that elicits aging. Furthermore, our study verifies the conservation of Co_3_O_4_ NPs' effect in activating UPR^mt^ and providing protection in mammalian cell systems. Taken together, the data suggest that Co_3_O_4_ NPs can function as a simple and efficient UPR^mt^ activator, laying the foundation for future development of potential cobalt-based nanomedical tools for therapeutics in anti-aging health care treatments.

## Methods

*Reagents and Strains:* Co(NO_3_)_2_·6H_2_O and aqueous ammonia was purchased from Sigma (St. Louis, MO). Dimercapto succinic acid (DMSA) was provided by Shanghai Lingfeng Chemical Reagent Co. Ltd. The DMSA modified Co_3_O_4_ NPs were synthesized by a coprecipitation method as previously introduced 27. In brief, Co(NO_3_)_2_·6H_2_O (0.291 g) and aqueous ammonia (0.81 mL, 25%) were mixed together under stirring at 60 °C (1000 rpm) for 3 h to obtain [Co(NH_3_)_6_]^3+^ solution. The obtained Co_3_O_4_ precipitates were suspended and mixed with dimercapto succinic acid (DMSA, 0.045 g in 1 mL DMSO) for sonication for 2 h and stirred for 5 h. The precipitates were collected again via centrifugation and washed three times before being dissolved in 50 mL of water. The mixture's pH was adjusted to 9.0-10.0, and then the mixture was sonicated to obtain a stable and clear solution. Finally, the solution was dialyzed in distilled water for 3 days to separate excess DMSA from Co_3_O_4_ NPs. The Co_3_O_4_ NPs dispersion was filtered through a 0.22 μm membrane and finally stored at 4 °C for the following studies. Fe_2_O_3_ NPs, Mn_3_O_4_ NPs and Prussian blue NPs were purchased from Nanjing Nanoeast Biotech co., LTD. ATP Assay Kit, 2′,7′-dichlorodihydrofluorescein diacetate (DCFH-DA) were purchased from Beyotime (Shanghai, China). D-galactose (Cat. No: G5388) was purchased from Sigma-Aldrich (St. Louis, MO). The compound of 5-hydroxy-1,4-naphthoquinone (Juglone, Sigma-Aldrich) was used to induce reactive oxygen species as an oxidative stress generator 49. Ethanol was adopted as a cosolvent of juglone. 5-fluoro-2-deoxyuridine (FUDR) was used to inhibit nematodes from laying eggs. Cell counting kit 8 (CCK8) assay kit was obtained from Dojindo (Kumamoto, Japan).

*C. elegans and Cells Maintenance:* Wild-type strains were provided by the Caenorhabditis Genetics Center (University of Minnesota). SJ4100 (zcIs13 [HSP-6::GFP]) was a general gift from Professor Caishiqing (Institute of Neuroscience and State Key Laboratory of Neuroscience, Chinese Academy of Sciences). Transgenetic worms SJ4103 (zcIs14 [myo-3::GFP(mit)]) that express mitochondrial-targeted pmyo-3::mito::GFP, as unrestricted gifts from Dr Chen Wenliang (Huainan Normal University), were used to monitor mitochondrial network morphology and contents. PS3551 [hsf-1(sy441)I] and FX0259 (dve-1) mutant worms were generally granted by Professor Xiaochen Wang (Institute of Biophisics, Chinese Academy of Sciences). Unless otherwise specified. The N_2_ Bristol strain was used as the reference wild type. *C. elegans* strains were cultured on the nematode growth medium (NGM) at 20 °C and maintained with standard procedures as described previously 50. Nematodes were obtained by bleaching gravid adults and allowing them to hatch overnight. Synchronized L1 larvae were placed on NGM plates spotted with Escherichia coli strain OP50. Upon reaching L4 stage, worms were transferred to NGM plates spotted with OP50, FudR (80 μM) and Co_3_O_4_ NPs or CoCl_2_ at indicated concentrations at 20 °C until further testing or observation. The HEK293T cell line was obtained from the ATCC cell bank and cultured in DMEM medium supplemented with 10% fetal bovine serum. Except otherwise indicated, cells were pretreated with Co_3_O_4_ NPs or CoCl_2_ at 0.05 μg mL^-1^ for 24 h before further tests.

*Characterization of Co_3_O_4_ NPs:* Transmission electron microscopy (TEM) was used to characterize the morphology and size of the Co_3_O_4_ NPs. The Co_3_O_4_ NPs solution was dropped onto carbon-coated copper grids and captured for TEM pictures (Jeol 2010, 200KV). Nanosizer ZS90 (Malvern) was leveraged to characterize Dynamic lighting scattering (DLS) and zeta potential distribution of Co_3_O_4_ NPs. Moreover, powder X-ray diffraction (XRD) data were collected on a Brueck D8 advance by using Cu Ka radiation. The diffractometer was operated at 40 kV and 40 mA.

*Lifespan Test:* Synchronized L1 larvae were placed on NGM plates spotted with OP50. Upon reaching L4 stage, approximately 50-100 synchronized L4 stage worms were transferred to NGM placed with OP50, FUDR (80 μM) and tested materials at 20 °C. In all lifespan experiments, worms were scored for live versus dead twice a day by gently tapping worms with a platinum wire. Worms that failed to respond to several taps were scored as dead and removed from the plate. Worms were censored if they died because of vulval rupture or desiccation from moving off the culture plate.

*Body Size Measurements:* Synchronized L1 larvae were placed on NGM placed with OP50, 5-FUDR (80 μM) and tested materials at 20 °C. 10-15 worms at indicated developmental age were randomly picked and imaged using a Leica M 165 FC dissecting microscope (Leica). Body size was determined by measuring the length of full body outline using ImageJ software.

*Motility Assay:* Synchronized L4 stage worms were placed on OP50 plates with FUDR and tested materials at 20 °C until the tested days. Individual nematodes were tracked and 30 s of continuous videos were recorded. The videos were analyzed to calculate worm thrashing number and body bend rate 50, 51. About 10-15 worms were calculated per group.

*Egg-laying rate*: A timed egg-lay was performed to synchronize populations. The L1 stages worms were treated with Co_3_O_4_ NPs (0.005-5 μg mL^-1^) for three days and collected on adult day 0. After exposure, 30 animals from each exposure group were transferred to new NGM agar plates and allowed to lay eggs for 2 h. Egg-laying rate was defined as the average total number of eggs (10 worms) per hour from the spawning period 52.

*Stress Resistance Assay:* A timed egg-lay was performed to synchronize populations. The L1 stages worms were treated with Co_3_O_4_ NPs for three days and collected on adult day 0. For the oxidative sensitivity assay, Co_3_O_4_ NPs and blank treated worms were seeded on NGM plates with 350 μM juglone and worm viability was monitored every 2 h until death of all worms tested. The number of dead worms was counted by provoking; The heat resistance performance of nematodes was examined 53. The pretreated worms were incubated at 35 °C for 3 h, followed by being deprived of food at 20 °C for 30 min. Then, the body bends and head thrashes were analyzed according to the procedures introduced above.

*Pumping Rate Assay*: We counted the contraction number per 30 seconds of the pharyngeal terminal bulb of Co_3_O_4_ NPs, CoCl_2_ or mock-treated worms on an OP50 bacterial lawn under a dissection scope, as described previously 54. One contraction of the posterior bulb/grind defines one pump. About 10 worms were calculated per group.

*Microscopy Analysis:* Random number of Co_3_O_4_ NPs, CoCl_2_ or mock-treated worms were placed on 2 % agarose pads and anesthetized with 20 mM NaN_3_ before the observation by fluorescence microscopy. To avoid the side effects of NaN_3_ on autophagy, all worm slides were freshly constructed and imaged within 15 min. Lipofuscin fluorescence images (excitation: 365 nm; emission: 420 nm) of the worms were captured densitometrically. For the strain SJ4103 (zcIs14 [myo-3::GFP(mit)]), the body region was captured using 63×CONFOCAL microscopy. Approximately 10-15 animals were captured for each experimental condition. Fiji ImageJ software was used to visualize and analyze the collected pictures.

*Cell Electron Microscopy Analysis:* HEK293T cells pretreated with or without Co_3_O_4_ NPs for 24 h as mentioned above were collected according to manufacture′s procedure. Electron microscopy was used to examine the ultrastructure of mitochondrial morphology.

*RNA Interference Experiments:* RNAi knockdown experiments were performed as previously described 54, 55. OP50 bacteria with plasmid clones targeting *atfs-1* and *ubl-5* were purchased from Shanghai HEWU BiotechnologyCo. LTD. The bacteria were cultured in LB culture medium containing 100 μg mL^-1^ carbenicillin overnight. Then the collected OP50 bacteria were placed on NGM plates together with carbenicillin (25 μg mL^-1^), isopropyl β-d-1-thiogalactopyranoside (IPTG, 1 mM), and 2′-deoxy-5-fluorouridine (FUDR, 80 μM). The OP50 bacteria carrying the empty vector L4440 were examined as the control group. Approximately 100 synchronized nematodes were placed on RNAi agar plates at the L4 stage, followed by test procedures.

*Worm ATP Level Quantification*: Worm ATP level quantification was performed according to a previously reported method 53. Approximately 100 pretreated nematodes on adult day 1 and day 7 were collected and washed three times to remove bacteria. Then resuspended worms were treated with 5 times freeze/thaw cycles (from liquid nitrogen to 40 °C) followed by boiling for 20 min. Then the cooled worm pellets were centrifuged at 11,000 × g for 10 min at 4 °C. The supernatant was used for protein quantification and ATP quantification using an ATP detection kit (Thermo Fisher, A22066).

*Oxygen Consumption Measurement*: We leveraged Seahorse XF24 equipment (Seahorse Bioscience) to measure worm oxygen consumption rate (OCR) 56. Specifically, synchronized worms were pretreated with or without Co_3_O_4_ NPs on adult day 1, day 4 and day 7. We rinsed the worms off the culturing plates and washed them three times with M9 buffer by gravity separation. Then, the samples were pipetted into wells of 24-well standard Seahorse assay plates (18 worms well^-1^), and 0.5 mL M9 buffer was placed into the blank well for oxygen consumption measurement. We set the instrument protocol to enable the mix cycle to 1, the wait cycle to 3, and the measure cycle to 3 min. Basal respiration was measured for 4 times. Sequentially, 20 μM FCCP (Sigma-Aldrich, Cat# C2920) was added and measured for 8 times to account for maximal respiration. Then 40 mM NaN_3_ was added and measured for 4 times to account for non-mitochondrial respiration. The OCR value was normalized to the number of worms per well.

*ROS Level Detection:* The *C. elegans* were pretreated with Co_3_O_4_ NPs as previously described and collected. For whole-cell ROS quantification, worms were stained with ROS probe DCFH-DA (Molecular Probes, 25 μM) for 30 min; For mitochondrial ROS quantification, the samples collected by centrifugation were stained by MitoSox solution (5 μM) for 20 min. Then the worms were washed for five times using M9 buffer to eliminate the excess reagent and were placed into 96-well plates for measurement. Quantitative analysis was conducted at excitation/emission wavelengths of 485 and 520 nm.

*MDA Content Assay:* Synchronized L4 stage worms were pretreated with or without 0.05 μg mL^-1^ Co_3_O_4_ NPs as previously described and collected on adult day 7. To remove bacteria outside of the worms, worms were rinsed three times with M9 buffer. Then the nematodes were collected and broken up by sonication for Malondialdehyde (MDA) analysis using commercially available kits* (*Beyotime Biotechnology, China) according to the manufacturer's instructions. The total protein mass in different was determined by BCA Protein Assay Kit (Beyotime Biotechnology, China).

*Iron quantitation*: Synchronized L4 stage worms were pretreated with or without 0.05 μg mL^-1^ Co_3_O_4_ NPs as previously described and collected on adult day 7. Then the dry weight of nematodes in each group was recorded before broken up by sonication. Total iron was measured using a 7700 Series (Agilent) inductively coupled plasma mass spectrometry (ICP-MS) as previously reported 57. Samples were consisted of at least 100 aged worms per replicate for each group.

*Bacteria growth rate:* To study if Co_3_O_4_ NPs exert any possible growth inhibitory and anti-proliferative effect on OP50 bacterial, 1-500 dilution cultures of OP50 bacteria was cultured in liquid LB in the presence of different concentrations of Co_3_O_4_ NPs in 15 mL sterile tubes. Then the OP50 bacteria were grown overnight with shaking at 37 °C. Absorbance (OD 595 nm) was measured at different time points using a microplate reader.

*Cell viability assessment:* Cell viability was determined using a cell counting kit 8 (CCK8) assay. HEK293T cells were seeded in 96-well tissue culture plates at a density of 1 × 10^4^ L^-1^ overnight. Then cells were treated with 0.05 μg mL^-1^ Co_3_O_4_ NPs in DMEM for 6 h. After that, cells were cultured in DMEM with 20 g L^-1^ D-galactose for 48 h to establish cellular aging model 48. Finally, 100 μL DMEM with 10% CCK8 was added into each well for 45 minutes incubation. The cell viability was determined by measuring optical absorbance at 450 nm. The assay was repeated in twice.

*RT-qPCR:* Total RNA was isolated from collected *C. elegans* using TRIzol (Invitrogen). DNA was wiped off using RQ1 RNase-Free DNase (Promega #M6101). cDNA was synthesized using the M-MLV Reverse Transcriptase (Invitrogen #28025013). Gene expression levels were determined by real-time PCR using SYBR Green Supermix. For* C. elegans*, the ratio of relative gene expression values for *nd-1* versus *act-3* represents mtDNA per nuclear genome. This was confirmed with a second mitochondrial gene *mtce.26* versus *act-3.* For mammalian HEK293T cells. HK2 was used as endogenous control for nuclear DNA and 16s was used as marker for mitochondrial DNA. All primers used in this study are listed in Table S1. All data were normalized to the control using actin.

*Western Blotting*: Western blotting analysis were performed with anti-ATP5A (Abcam), anti-MTCO1 (Abcam), anti-CLPP (Proteintech), anti-SDHA (Proteintech) and anti-GAPDH (Santa Cruz Biotechnology) antibodies. For* C. elegans*, synchronous L4 worms were treated with or without Co_3_O_4_ at 0.05 μg mL^-1^ and collected on adult day 1; For mammalian HEK293T cells, cells were treated with indicated concentrations of Co_3_O_4_ NPs for 24 h. Then nematodes or cells were collected in lysis buffer were sonicated and centrifuged to obtain the supernatant. Afterwards, the samples were boiled in SDS sample buffer for 5-10 min, and separated on SDS-polyacrylamide gel elec trophoresis (Bio-Rad) by standard western blotting procedure 58.

*Statistical Analysis*: All statistics were performed using Graph Pad Prism 7 software. The results are presented as mean ± SE. Data were analyzed by one-way- or two-way analysis of variance (ANOVA), followed by Tukey's multiple comparisons posttest or log-rank test. Differences were regarded as statistically significant (*P < 0.05; **P < 0.01; ***P < 0.001).

## Supplementary Material

Supplementary figures and table.

## Abbreviations

UPR^mt^: mitochondrial unfolded protein response
Co_3_O_4_ NPs: cobalt oxide nanoparticles
mtDNA: mitochondrial DNA
nDNA: nuclear DNA
*C. elegans*: *Caenorhabditis elegans*
DMSA: dimercaptosuccinic acid
ROS: reactive oxygen species
DCFDA: 2′,7′-dichlorofluorescin diacetate
TEM: transmission electron microscopy
DLS: dynamic light scattering
UBL-5: ubiquitin-like protein-5
ATFS-1: activating transcription factor-1
HSF-1: heat shock factor-1
HSR: heat shock response
HSF-4: heat shock factor-4
ER: endoplasmic reticulum
*hxk-1*: hexokinase-1
*cox-4*: cytochrome *c* oxidase IV
*cts-1*: citrate synthase
*pyc-1*: pyruvate carboxylase*-1*
5-FUDR: 5-fluoro-2-deoxyuridine
NGM: nematode growth medium
XRD: x-ray diffraction
IPTG: isopropyl β-d-1-thiogalactopyranoside
OCR: oxygen consumption rate
HEK293T: human embryonic kidney cells
h: hours
CCK8: cell counting kit 8
ICP-MS: inductively coupled plasma mass spectrometry
MDA: malondialdehyde
