# Supplementary material for: Dietary cobalt oxide nanoparticles alleviate aging through activation of mitochondrial UPR in Caenorhabditis elegans
Source: Theranostics. 2023 May 27;13(10):3276–89. doi: 10.7150/thno.81817 (PMC10283066; doi:10.7150/thno.81817)
Supplement: Supplementary file 1 — Supplementary figures and table. [file thnov13p3276s1.pdf]

Supporting Information

Dietary cobalt oxide nanoparticles alleviate aging through activation of mitochondrial UPR in *Caenorhabditis elegans*

Wenshu Cong [1,2], Yajie Wang [3], Chunhui Yuan [2], Mei Xu [2], Han Wang [2], You Hu [2], Xuyan Dai [4], Yuhua Weng [1], Peter Timashev [5], Xing-Jie Liang [6], Yuanyu Huang [1,*]

[1] Advanced Research Institute of Multidisciplinary Science; School of Life Science; School of Medical Technology; Key Laboratory of Molecular Medicine and Biotherapy; Key Laboratory of Medical Molecule Science and Pharmaceutics Engineering; Beijing Institute of Technology, Beijing 100081, P. R. China.

[2] Jiangsu Key Laboratory of Brain Disease and Bioinformation, Research Center for Biochemistry and Molecular Biology, Xuzhou Medical University, Xuzhou 221004, P. R. China

[3] Key Laboratory of Combinatorial Biosynthesis and Drug Discovery, Ministry of Education, School of Pharmaceutical Sciences, Wuhan University, Wuhan 430071, P. R. China

[4] Hunan Agricultural University, Changsha 410128, P. R.China

[5] Laboratory of Clinical Smart Nanotechnologies, Institute for Regenerative Medicine, Sechenov University, 119991 Moscow, Russia

[6] CAS Key Laboratory for Biomedical Effects of Nanomaterials and Nanosafety, CAS Center for Excellence in Nanoscience, National Center for Nanoscience and Technology of China, Beijing 100190, P. R. China

* Corresponding author. E-mail: yyhuang@bit.edu.cn (Y.H.)

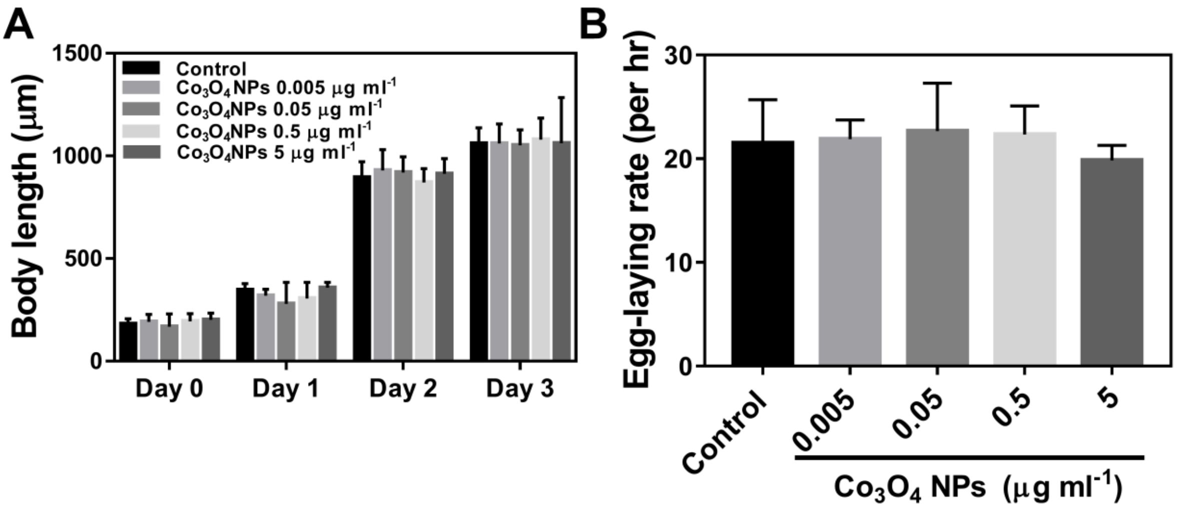

Figure S1. Biosafety assessment of Co_3O_4 NPs on *C. elegans*. Effects of Co_3O_4 NPs on worms were shown in body length (A) and egg-laying rate (B). Wild-type worms at L1 larva stage were treated with Co_3O_4 NPs with gradient doses (0.005-5 μg ml^{-1}) for three days. Bars represent means ± SD.

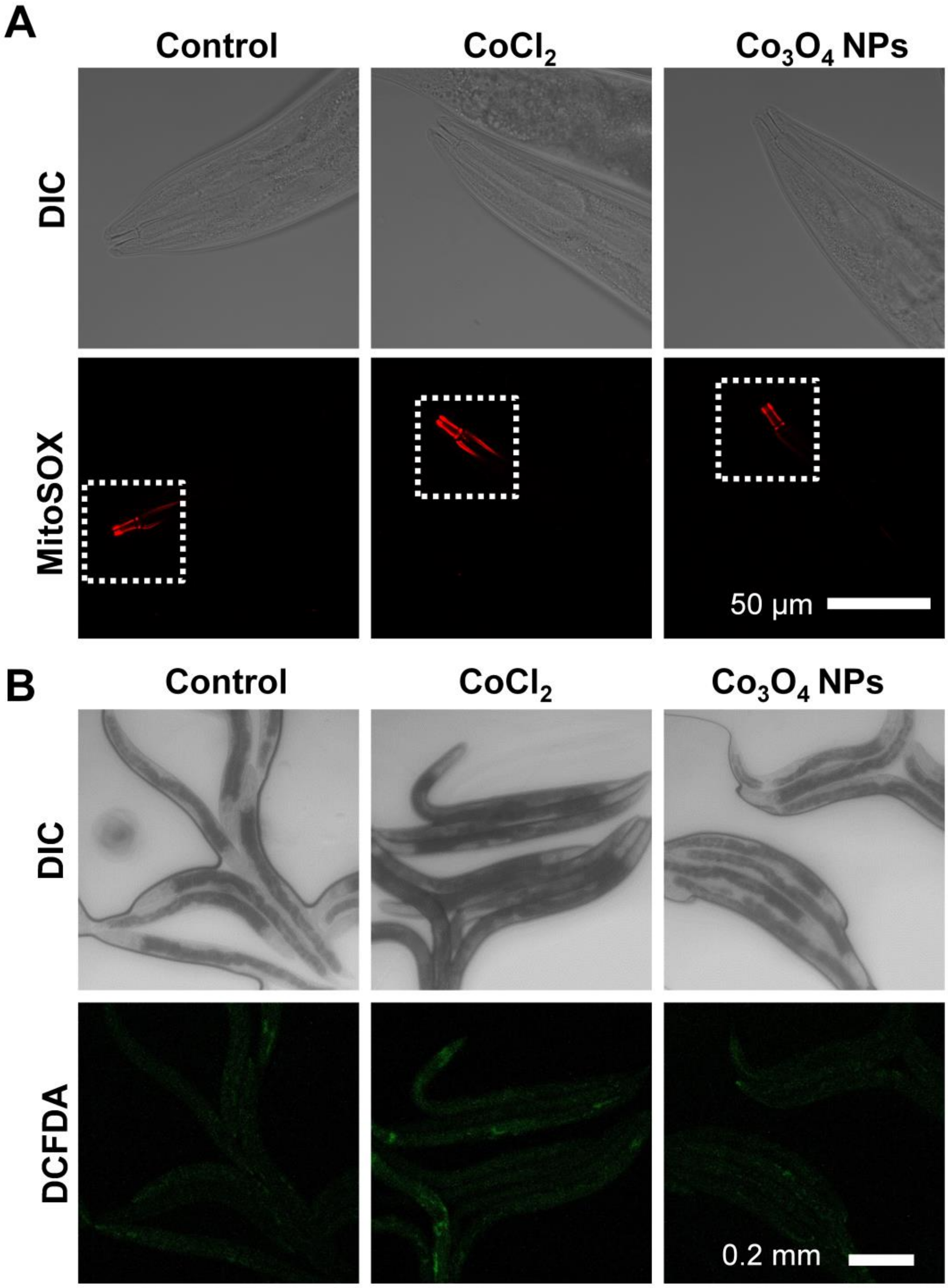

Figure S2. Representative pictures of mitochondrial ROS on nematode head (A) and cellular ROS (B) in Co_3O_4 NPs, $CoCl_2$ or mock-treated worms on adult day 1.

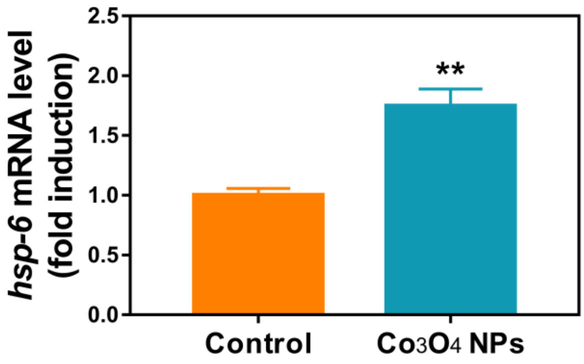

Figure S3. Influence of Co_3O_4 NPs on *hsp-6* (homologous gene of *hsp60*) expression. *mRNA* expression of *hsp-6* of worms on adult day 1 pretreated with Co_3O_4 NPs.

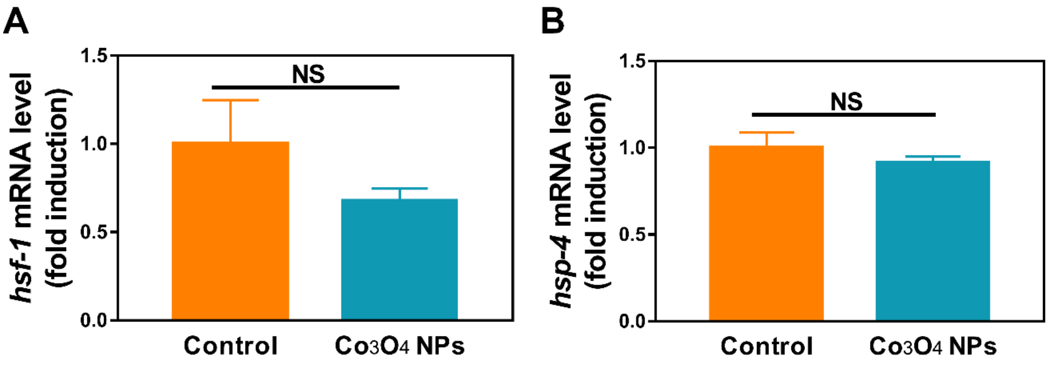

Figure S4. Influence of Co_3O_4 NPs on *hsf-1* and *hsp-4* expression. (A) Expression of *hsf-1* in worms on adult day 1 pretreated with Co_3O_4 NPs. (B) Expression of *hsp-4* in worms on adult day 1 pretreated with Co_3O_4 NPs..

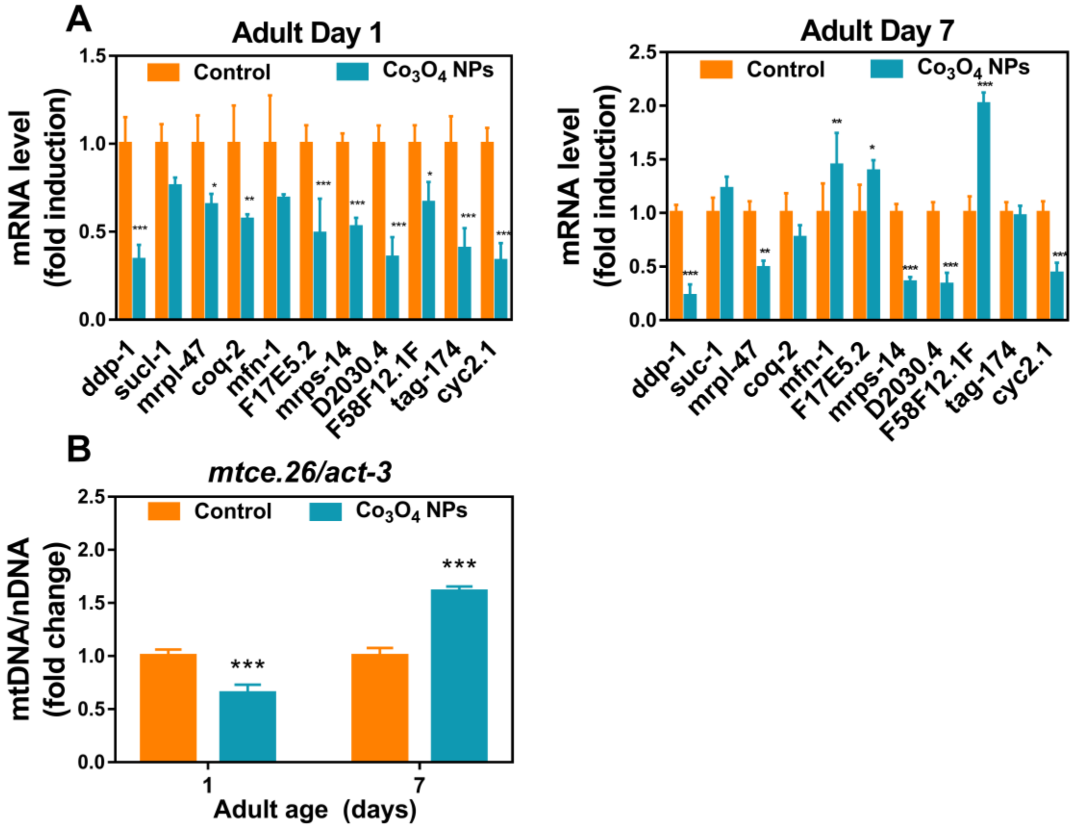

Figure S5. The effect of Co_3O_4 NPs on mitochondrial abundance. (A) RT-qPCR analysis of changes in the expression of nuclear genes encoding mitochondrial proteins in wild-type worms on adulthood days 1 and 7 pretreated with Co_3O_4 NPs. (B) RT-qPCR analysis of mtDNA/nDNA ratio (*mtce.26/ act-3*) in wild-type worms on adulthood days 1 and 7 pretreated with Co_3O_4 NPs.

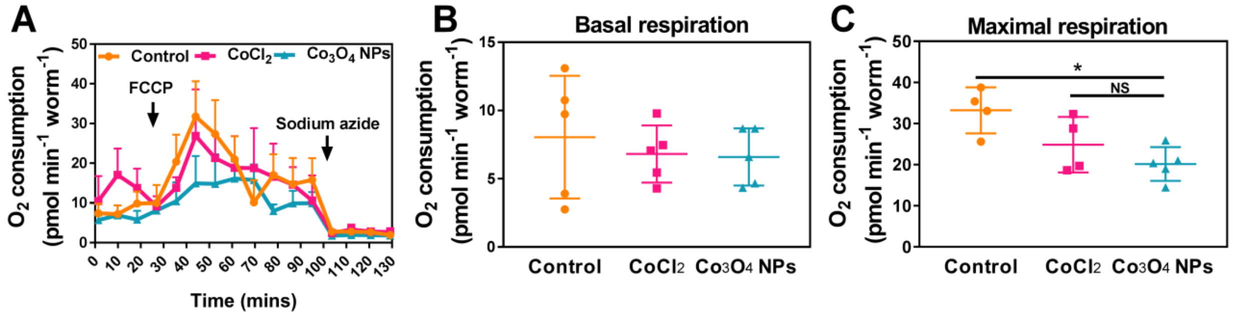

Figure S6. Influence of Co_3O_4 NPs on oxygen consumption rate (OCR) compared to $CoCl_2$ and negative control. (A) Oxygen consumption curves of Co_3O_4 NPs, $CoCl_2$ treated worms or blank control in the presence FCCP and NaN_3 on adult day 4. Comparison of the basal oxygen consumption rate (B) and maximum oxygen consumption rate (C) in N_2 wildtype worms treated with Co_3O_4 NPs or $CoCl_2$ on adult day 4.

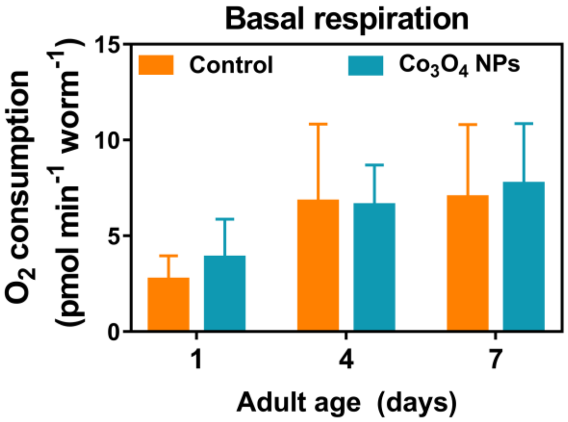

Figure S7. Basal oxygen consumption of N2 wildtype worms treated with Co_3O_4 NPs or not on adult days 1, 4 and 7.

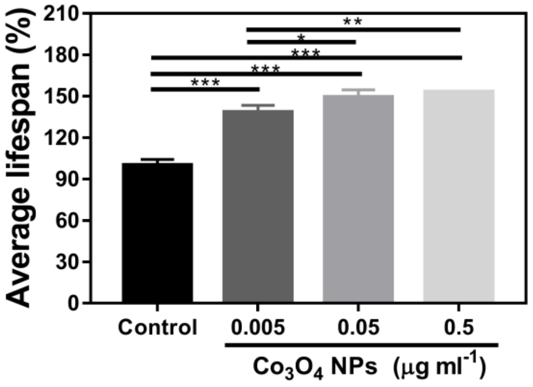

Figure S8. The average lifespan (%) of worms treated with Co_3O_4 NPs (from 0 to 0.5 μg ml^{-1}).

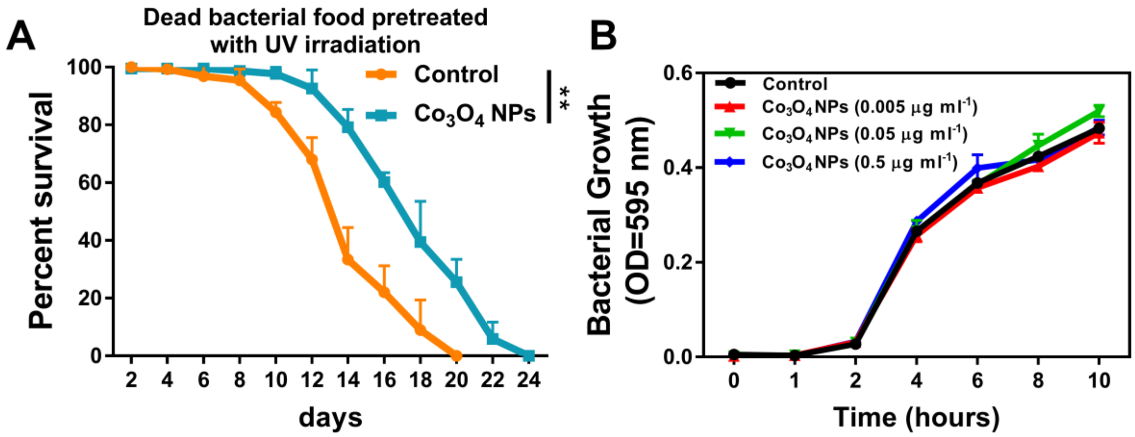

Figure S9. (A) Lifespan curves of *C. elegans* treated with Co_3O_4 NPs in dead bacterial food pretreated with UV irradiation for 30 minutes. (B) OP50 bacteria in liquid LB treated with different concentrations of Co_3O_4 NPs did not show growth retardation.

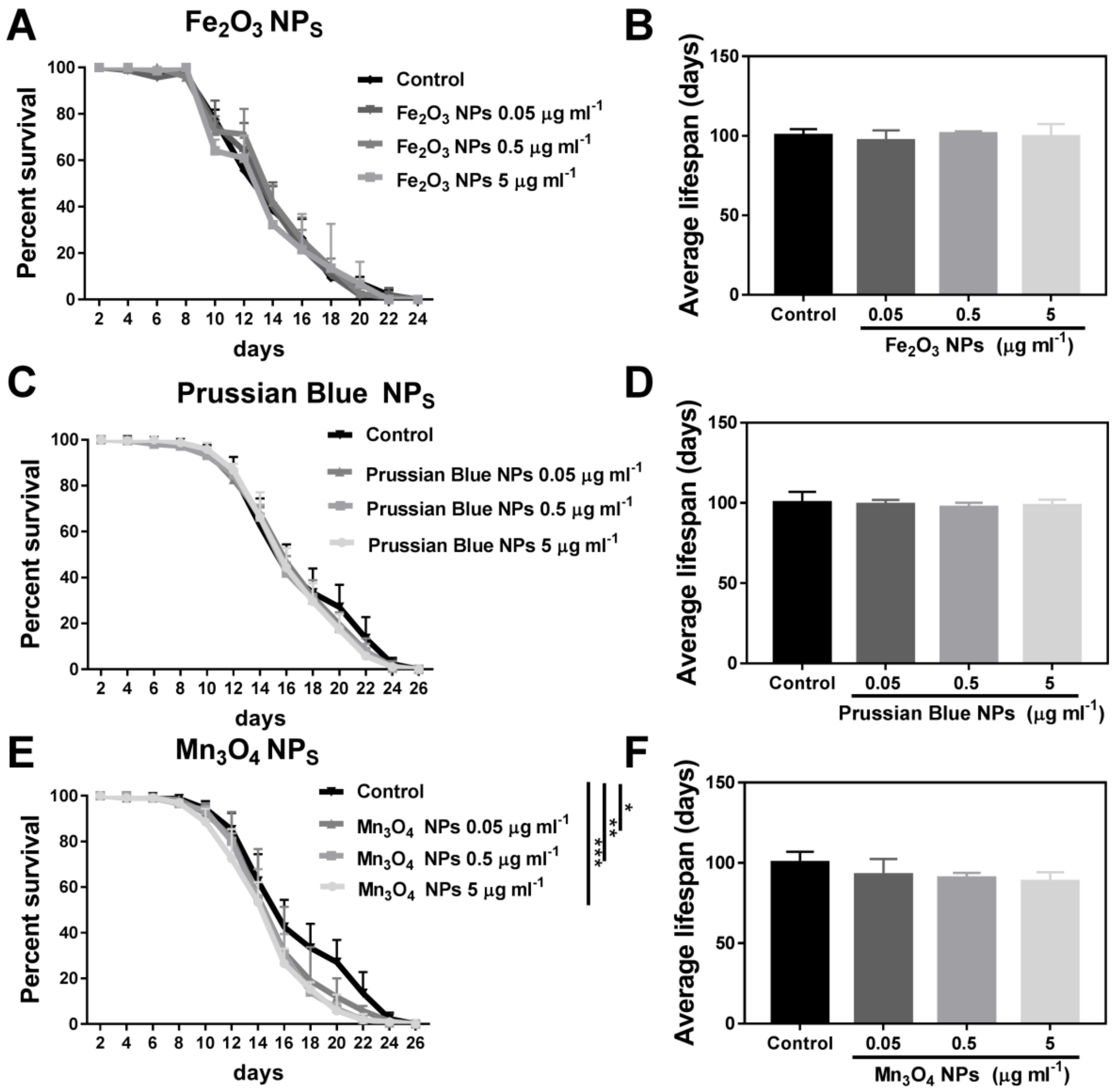

Figure S10. Influence of Fe_2O_3 NPs, Prussian Blue NPs and Mn_3O_4 NPs on lifespan of *C. elegans.* (A) Lifespan curves of *C. elegans* treated with Fe_2O_3 NPs. (B) The average lifespan (%) of worms treated with Fe_2O_3 NPs (from 0 to 5 μg ml^{-1}). (C) Lifespan curves of *C. elegans* treated with Prussian Blue NPs. (D) The average lifespan (%) of worms treated with Prussian Blue NPs (from 0 to 5 μg ml^{-1}). (E) Lifespan curves of *C. elegans* treated with Mn_3O_4 NPs. (F) The average lifespan (%) of worms treated with Mn_3O_4 NPs (from 0 to 5 μg ml^{-1}).

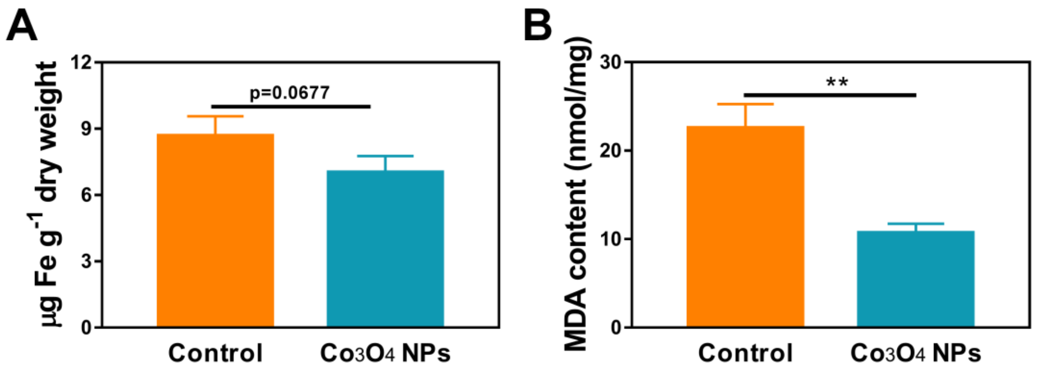

Figure S11. Influence of Co_3O_4 NPs on Fe accumulation and MDA content in *C. elegans*
A. *In vivo* Fe levels (μg Fe per dry weight measured via ICP-MS) in worms treated with or without Co_3O_4 NPs on adult day 7. B. Malondialdehyde (MDA) content in worms treated with or without Co_3O_4 NPs on adult day 7.

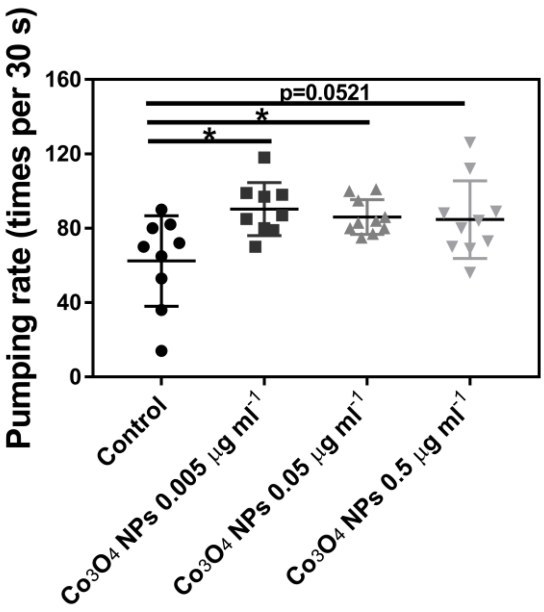

Figure S12. Pharyngeal pumping in worms treated with Co_3O_4 NPs at different doses on adult day 6.

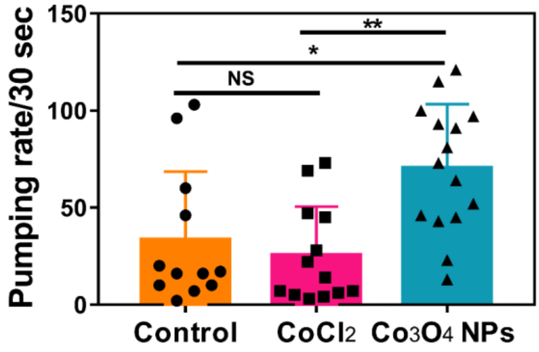

Figure S13. Pharyngeal pumping in worms treated with Co_3O_4 NPs or $CoCl_2$ at 0.05 µg ml^{-1} on adult day 9.

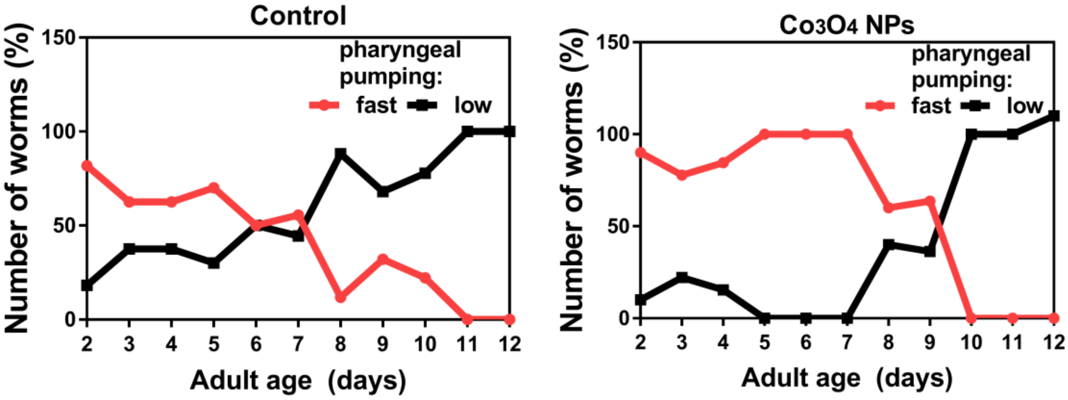

Figure S14. Co_3O_4 NPs' effect on pumping rate by quantifying the ratio of worms that are fast-, slow-pumpers. Typically, pharyngeal contractions were divided the following subgroups: <6 per minute (not pumping), 6-147 per minute (slow pumping), and >147 (fast pumping), respectively.

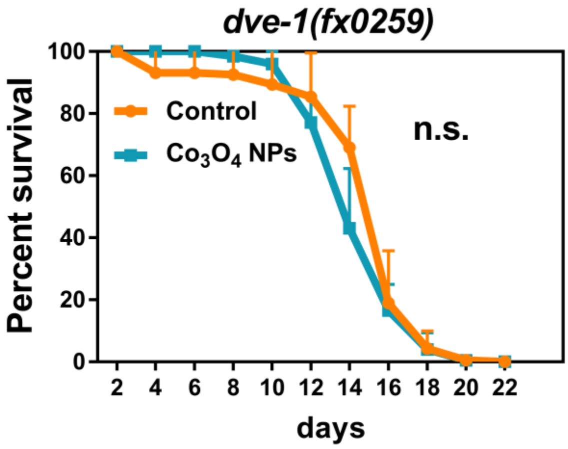

Figure S15. **Supplementation of Co_3O_4 NPs does not extend lifespan in the *dev-1(fx0259)***

mutant (n.s. = not significant).

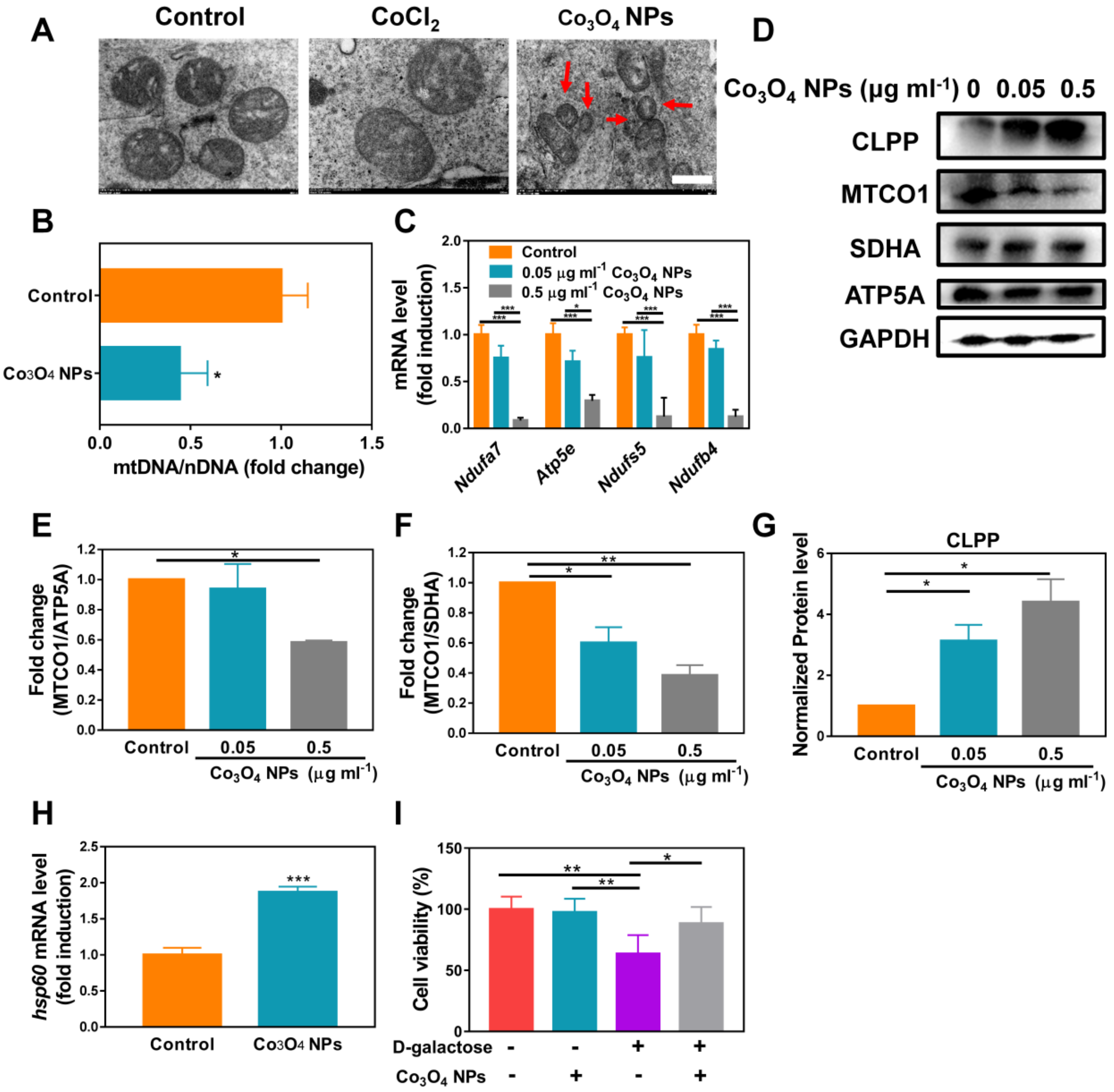

Figure S16. Effect of Co_3O_4 NPs on mitochondrial homeostasis in mammalian cells. (A) Mitochondrial morphology of cells treated with Co_3O_4 NPs, $CoCl_2$ or blank control by transmission electron microscopy. Mitochondrial in smaller size versus average was indicated by red arrows (B) Treatment with Co_3O_4 NPs decreases the ratio between mitochondrial DNA (mtDNA) and nuclear DNA (nDNA), a common marker for mitochondrial abundance in HEK293t cells. (C) Effects of Co_3O_4 NPs on the transcription of mitochondrial-related genes in HEK293t cells. (D) Western blots pics of CLPP, MTCO1, SDHA and ATP5A in HEK293t cells treated with or without Co_3O_4 NPs. GAPDH served as a loading control. (E) Quantitative analysis on the ratio of mtDNA-encoded MTCO1 and nDNA-encoded ATP5A in HEK293t cells treated with Co_3O_4 NPs. (F) Quantitative analysis on the ratio of mtDNA-encoded MTCO1 proteins and nDNA-encoded SDHA in HEK293t cells treated with Co_3O_4 NPs. (G) Quantitative

analysis on the UPR^{mt} protease CLPP expression in HEK293t cells treated with Co_3O_4 NPs. (H) The mRNA expression level of *hsp60* in HEK293t cells treated with Co_3O_4 NPs. (I) Effect of Co_3O_4 NPs on D-galactose-induced cellular viability decline. HEK293t cells were pretreated with Co_3O_4 NPs at 0.05 μg ml^{-1} in DMEM medium, and then exposed to D-galactose (20 g L^{-1}) for 48 h. CCK8 assay was used to measure cellular viability. Bar graphs are expressed as mean±SEM. *P<0.05, **P<0.01, ***P<0.001. Scale bar=5 μm.

Table S1. Primers used in RT-qPCR

Gene name	Sequence (5'-3')
cts-1-rv	GGTACAGGTTGCGATAGATGATAGC
cts-1-fw	CTCGACAACTTCCCAGATAACC
cox-4-rv	AGGTTGGCGGCAGTTCTGGG
cox-4-fw	GCCCCAATTCGCGCCAAGGA
hxk-1-rv	CTAGAGATGACGTCACACACTTCTC
hxk-1-fw	GTGCGACGAGTACTTTCTCAACTG
fzo-1-rv	AGTCGGCATTCCCCTGATTCCG
fzo-1-fw	TCTGCAGGTTGAAGGTTCAGAAGGC
opa-1-rv	CTTGGCCATCCATTCTGCCCA
opa-1-fw	TCGCGGCTAGAACGTGGTATGA
hsp-6-rv	AGCGATGATCTTATCTCCAGCGTCC
hsp-6-fw	AACCACCGTCAACAACGCCG
pyc-1-rv	GTGATCATACATCCTGGTCTACTGC
pyc-1-fw	TCCAACTACTCCTCTTGCTACTGAC
ddp-1-rv	AGTGCTCGACCATGAAGTTG
ddp-1-fw	AACAAGTGCACACGCTCA
sucl-1- rv	CAGCTGATCCTCCGATTTCT
sucl-1- fw	GTCGGATTCGGACAGACTTT
mrpl-47-rv	ACTCGTGGAGCTCCTCTCTTGA
mrpl-47-fw	CGACGACGATGCCTACGTGA
mrps-14-rv	TTGGACTCCACTGAGAGCTG

mrps-14-fw	CGATCATCCTCGTCTGATTC
coq-2-rv	CCGATGTTCTTCCTCGATTC
coq-2-fw	AGTCAAACTCATGGATGGGAA
mfn-1- rv	TGCAGGAACCTGGAATATGA
mfn-1-fw	GATCCAGCAAATAGGCGAAT
F17E5.2-rv	CCTTCATGAAATTCGGTGTG
F17E5.2- fw	TCGAACAAGGCTTCAAGCTA
F58F12.1-rv	TGATCAATGCTTCAGCAACTT
F58F12.1-fw	CTTGATGCTGCCCAAAGAG
D2030.4-rv	TTCAAGCAGTGGCTTCTTTG
D2030.4-fw	TGGAGCTTGGGATAAATGTG
tag-174-rv	TAGTGCTTCTCACGGTCAGC
tag-174-fw	TCGAGTACGCCTTCTTGAAC
cyc-2.1-rv	TTCTTGAGTCCAGCGAACAC
cyc-2.1-fw	CGCTGCTAACAAGAACAAGG
act-1-rv	GTAGCAGAGCTTCTCCTTGATGTC
act-1-fw	GCTGGACGTGATCTTACTGATTACC
act-3-rv	GGTGGTTCTCCGGAAAGAA
act-3-fw	TGCGACATTGATATCCGTAAGG
nd-1-rv	AAGCTTGTGCTAATCCCATAAATGT
nd-1-fw	AGCGTCATTTATTGGGAAGAAGAC
mtce.26-rv	CAGGGTGCCCCATTGTTCTT
mtce.26-fw	GGTTGTGGGACTAGGTGAACA
HK2- rv	GGGAACACAAAAGACCTCTTCTGG
HK2- fw	GCCAGCCTCTCCTGATTTTAGTGT
16s-rv	TCGTTTGGTTTCGGGGTTTC
16s-fw	CCGCAAGGGAAAGATGAAAGAC
Ndufa7-rv	GAGCTTGTGGCTAGGACCC
Ndufa7-fw	TGCAGCTACGCTACCAGGA
Ndufs5-rv	CCGAAGCAAACACTCTACGAAAT

Ndufs5-fw	TGCACATGGAATCGGTTATACTC
Ndufb4-rv	GAAGCAGGTACTCTCGTTTCAG
Ndufb4-fw	ATGTCGTTCCCAAAGTATAAGCC
Atp5e-rv	GGAGTATCGGATGTAGCTGAGT
Atp5e-fw	GTGGCCTACTGGAGACAGG
hsp60-rv	AGCCCGAGTGAGATGAGGAG
hsp60-fw	ATGCTTCGGTTACCCACAGTC
